# Molecular Scanning and Morpho-Physiological Dissection of Component Mechanism in *Lens* Species in Response to Aluminium Stress

**DOI:** 10.1371/journal.pone.0160073

**Published:** 2016-07-28

**Authors:** Dharmendra Singh, Madan Pal, Chandan Kumar Singh, Jyoti Taunk, Priyanka Jain, Ashish Kumar Chaturvedi, Sadhana Maurya, Sourabh Karwa, Rajendra Singh, Ram Sewak Singh Tomar, Rita Nongthombam, Nandini Chongtham, Moirangthem Premjit Singh

**Affiliations:** 1 Division of Genetics, Indian Agricultural Research Institute, New Delhi, India; 2 Division of Plant Physiology, Indian Agricultural Research Institute, New Delhi, India; 3 Division of Soil Science and Agricultural Chemistry, Indian Agricultural Research Institute, New Delhi, India; 4 National Research Centre on Plant Biotechnology, Indian Agricultural Research Institute, New Delhi, India; 5 KVK West Siang, ICAR RC for NEH Region, A.P. Centre, Basar, Arunachal Pradesh, India; 6 KVK Imphal East, Andro, Central Agricultural University, Imphal, Manipur, India; 7 Directorate of Extension Education, Central Agricultural University, Imphal, Manipur, India; National Institute of Plant Genome Research (NIPGR), INDIA

## Abstract

Aluminium (Al) stress was imposed on 285 lentil genotypes at seedling stage under hydroponics to study its effects on morpho-physiological traits where resistant cultigens and wilds showed minimum reduction in root and shoot length and maximum root re-growth (RRG) after staining. Molecular assortment based on 46 simple sequence repeat (SSR) markers clustered the genotypes into 11 groups, where wilds were separated from the cultigens. Genetic diversity and polymorphism information content (PIC) varied between 0.148–0.775 and 0.140–0.739, respectively. Breeding lines which were found to be most resistant (L-7903, L-4602); sensitive cultivars (BM-4, L-4147) and wilds ILWL-185 (resistant), ILWL-436 (sensitive) were grouped into different clusters. These genotypes were also separated on the basis of population structure and Jaccard’s similarity index and analysed to study Al resistance mechanism through determination of different attributes like localization of Al and callose, lipid peroxidation, secretion of organic acids and production of antioxidant enzymes. In contrast to sensitive genotypes, in resistant ones most of the Al was localized in the epidermal cells, where its movement to apoplastic region was restricted due to release of citrate and malate. Under acidic field conditions, resistant genotypes produced maximum seed yield/plant as compared to sensitive genotypes at two different locations *i*.*e*. Imphal, Manipur, India and Basar, Arunanchal Pradesh, India during 2012–13, 2013–14 and 2014–15. These findings suggest that Al stress adaptation in lentil is through exclusion mechanism and hybridization between the contrasting genotypes from distinct clusters can help in development of resistant varieties.

## Introduction

Globally, lentil is cultivated on 4.5 million ha with a production of 4.9 million tons [1]. The major lentil-growing countries include India, Canada, Turkey, Syria, Australia, Nepal and United States. These countries have large acreage under acidic soils with a problem of aluminium (Al) toxicity [2]. Al toxicity affects 40–70% of the world arable lands that causes 25–80% yield losses in crops grown on soils containing excessive Al [3, 4]. In soils, Al is found in the form of insoluble alumina-silicates or oxides. When pH of the soil drops below 5.0, Al is solubilised in the form of phytotoxic Al^3+^ ions which becomes toxic for many crops. Among various Al toxicity symptoms, the first visible symptom is rapid inhibition of root growth which can occur within hours or even minutes of exposure to Al^3+^ [5]. This trait has been used as a biomarker to estimate Al sensitivity in crop plants. Although, Al toxicity can be reduced through application of lime which raises the soil pH, however, this amendment does not bring permanent remedy to sub-soil acidity. Further, liming may not always be practical and is not a cost effective method. Alternatively, the most appropriate strategy to overcome Al toxicity is to grow resistant varieties. In order to explore sources of resistance, it is necessary to screen a large number of genotypes by using reliable and effective screening techniques. Two most reliable screening parameters are root re-growth (RRG) after staining and callose accumulation in roots under hydroponic assay [2, 6, 7]. This assay has unconditional advantages over field screening, as Al concentration in soil may not always be uniform and other environmental factors may interact with soil Al to mask the expression of Al resistance [8]. However, screening for Al resistance under hydroponics in combination with field evaluation for Al resistance would be the best approach for characterization of whole plant resistance in terms of seed yield and its contributing traits.

Molecular diversity is the key pillar of diversity within species, between species and of ecosystems [9]. This diversity can be exploited for development of varieties resistant to different abiotic stresses through molecular breeding. Molecular analysis of genotypes and physiological mechanism for Al resistance has been studied in many legumes like common bean, chickpea etc. [10, 11], but to our knowledge no such study has been done in case of *Lens* species so far. SSRs are reliable marker system for molecular studies with attributes like robustness, high level of polymorphism, high reproducibility, co-dominance etc. These markers have been used in lentil to study diversity in relation to many abiotic stresses like drought, heat etc. and can be successfully utilized for diversity analysis for Al resistance as well [12, 13].

Callose induction in roots has been identified as a reliable physiological trait for detection of Al toxicity and is believed to mediate by Al^3+^ stress even under short-term exposure to Al [6, 7, 14]. Also, production of reactive oxygen species (ROS) has been reported under Al stress environments [15]. ROS accumulation in cells leads to lipid peroxidation and membrane damage and affects various cellular activities [16]. To counter oxidative damage caused by ROS production, plants have developed various enzymatic and non-enzymatic antioxidant systems [17]. Increased antioxidative enzyme activities facilitate the removal of excessive ROS and check lipid peroxidation [18, 19]. Main enzymes involved in the homeostatic control of ROS in plant systems are superoxide dismutase (SOD; EC 1.15.1.1), catalase (CAT; EC 1.11.1.6), ascorbate peroxidase (APX; EC 1.11.1.11) and guaiacol peroxidase (GPX; EC 1.11.1.7) [17]. These enzymatic systems have been used as indicators of resistance to Al in some crop plants [17]. The potential mechanisms conferring resistance to Al^3+^ can be broadly divided into those that exclude Al^3+^ from the root symplast (exclusion mechanisms) and those that enable plants to cope with Al^3+^ safely, once it enters the symplast (tolerance mechanism) [20]. The mechanisms conferring resistance to Al toxicity have been focussed in many crop plants [5, 21, 22, 23], but no such study has been reported in lentil so far. Therefore, the present investigation was planned to: (i) evaluate aluminium resistance among genotypes based on morpho-physiological traits ii) assort lentil genotypes with different adaptations to aluminium stress conditions for their genetic variation using SSRs and iii) reveal component mechanisms for Al resistance in lentil.

## Materials and Methods

### Plant materials

Two hundred eighty five genotypes representing all the subspecies of the genus *Lens*; cultivated *Lens culinaris* Medikus ssp. *culinaris* including breeding lines, landraces, germplasm collections and cultivars along with the wild species *viz*. *L*. *culinaris* ssp. *orientalis* (Boiss.) Ponert, *L*. *culinaris* ssp. *odemensis* (Ladiz), *L*. *nigricans* M. B. Godr. ssp. *nigricans* Godr., *L*. *nigricans* ssp. *ervoides* (Brign.) Ladiz., *L*. *lamottei* Czefr. originating from 22 different locations (origin of 5 genotypes unknown) *viz*. ICARDA (132), India (66), Turkey (26), Syria (24), Israel (4), Bangladesh, Italy, Jordan and Spain (3 each); Argentina, France, Lebanon and Slovenia (2 each); Azerbaijan, Croatia, Ethiopia, Mexico, Nepal, Palestine, Tajikistan and Uzbekistan (1 each) were used for evaluation of Al resistance (Table 1).

**Table 1:** Genotypes with different origins, type and sensitivity to Al stress (AR).

S. No.	Genotype	Origin	Type	AR	S.No.	Genotype	Origin	Type	AR
1	121–12	India	GC	MR	144	ILL-590	Turkey	GC	MR
2	1220–11	India	BL	MR	145	ILL-6002	ICARDA	GC	R
3	210–11	India	BL	MR	146	ILL-7349	Nepal	GC	MR
4	330–12	India	GC	MR	147	ILL-76037	ICARDA	GC	MR
5	BM-4	Bangladesh	Cult.	S	148	ILL-7978	ICARDA	GC	MR
6	DPL-62	India	Cult.	MR	149	ILL-7979	ICARDA	GC	MR
7	E-153	India	GC	MR	150	ILL-7982	ICARDA	GC	MR
8	FLIP-96-51	ICARDA	GC	MR	151	ILL-8006	Bangladesh	GC	MR
9	IG-109039	ICARDA	GC	S	152	ILL-8108	Argentina	GC	MR
10	IG-111991	ICARDA	LR	MR	153	ILL-8329	ICARDA	GC	MR
11	IG-111996	ICARDA	LR	MR	154	ILL-91887	ICARDA	GC	MR
12	IG-112078	ICARDA	LR	MR	155	ILL-9841	ICARDA	GC	MR
13	IG-11210	ICARDA	LR	MR	156	ILL-9900	ICARDA	GC	MR
14	IG-112128	ICARDA	LR	S	157	ILL-9916	ICARDA	GC	MR
15	IG-112131	ICARDA	LR	MR	158	ILL-9941	ICARDA	GC	MR
16	IG-112137	ICARDA	LR	MR	159	ILL-9960	ICARDA	GC	MR
17	IG-116551	ICARDA	LR	MR	160	ILWL-06	Turkey	Wild	MR
18	IG-129185	ICARDA	LR	MR	161	ILWL-09	Syria	Wild	MR
19	IG-129214	ICARDA	LR	MR	162	ILWL-10	-	Wild	MR
20	IG-129287	ICARDA	LR	MR	163	ILWL-100	Turkey	Wild	MR
21	IG-129291	ICARDA	LR	MR	164	ILWL-104	Turkey	Wild	MR
22	IG-129293	ICARDA	LR	MR	165	ILWL-125	Syria	Wild	S
23	IG-129302	ICARDA	LR	S	166	ILWL-128	Syria	Wild	MR
24	IG-129309	ICARDA	LR	MR	167	ILWL-13	Italy	Wild	MR
25	IG-129313	ICARDA	LR	MR	168	ILWL-133	Syria	Wild	MR
26	IG-129315	ICARDA	LR	MR	169	ILWL-137	Syria	Wild	MR
27	IG-129317	ICARDA	LR	MR	170	ILWL-142	Syria	Wild	MR
28	IG-129319	ICARDA	LR	R	171	ILWL-15	France	Wild	MR
29	IG-129372	ICARDA	LR	MR	172	ILWL-165	Syria	Wild	MR
30	IG-129560	ICARDA	LR	MR	173	ILWL-184	Syria	Wild	MR
31	IG-12970	ICARDA	LR	MR	174	ILWL-185	Syria	Wild	R
32	IG-130033	ICARDA	LR	MR	175	ILWL-192	Syria	Wild	MR
33	IG-130219	ICARDA	LR	S	176	ILWL-20	Palestine	Wild	MR
34	IG-130272	ICARDA	LR	MR	177	ILWL-203	Turkey	Wild	MR
35	IG-134342	ICARDA	LR	MR	178	ILWL-221	Turkey	Wild	MR
36	IG-134347	ICARDA	LR	MR	179	ILWL-227	Syria	Wild	S
37	IG-134356	ICARDA	LR	MR	180	ILWL-23	Italy	Wild	MR
38	IG-135424	-	Wild	MR	181	ILWL-237	Syria	Wild	MR
39	IG-135428	-	Wild	MR	182	ILWL-238	Syria	Wild	MR
40	IG-136607	ICARDA	LR	MR	183	ILWL-253	Syria	Wild	MR
41	IG-136608	-	Wild	MR	184	ILWL-269	Turkey	Wild	MR
42	IG-136612	Turkey	Wild	MR	185	ILWL-29	Spain	Wild	R
43	IG-136614	Italy	Wild	MR	186	ILWL-292	Turkey	Wild	MR
44	IG-136618	Croatia	Wild	MR	187	ILWL-3	Turkey	Wild	R
45	IG-136620	Slovenia	Wild	S	188	ILWL-314	Turkey	Wild	MR
46	IG-136626	Israel	Wild	MR	189	ILWL-320	Turkey	Wild	MR
47	IG-136637	France	Wild	MR	190	ILWL-321	Turkey	Wild	MR
48	IG-136652	Israel	Wild	MR	191	ILWL-334	Jordan	Wild	MR
49	IG-136653	Israel	Wild	MR	192	ILWL-377	Tajiskistan	Wild	MR
50	IG-136673	Turkey	Wild	MR	193	ILWL-340	Jordan	Wild	R
51	IG-136788	Syria	Wild	MR	194	ILWL-35	Turkey	Wild	MR
52	IG-140910	Azerbaijan	Wild	MR	195	ILWL-350	Syria	Wild	MR
53	IG-149	ICARDA	LR	MR	196	ILWL-357	Syria	Wild	MR
54	IG-129304	ICARDA	LR	MR	197	ILWL-361	Syria	Wild	MR
55	IG-49	ICARDA	LR	MR	198	ILWL-362	Syria	Wild	MR
56	IG-5320	ICARDA	LR	S	199	ILWL-366	Syria	Wild	MR
57	IG-69540	ICARDA	LR	MR	200	ILWL-370	Syria	Wild	MR
58	IG-69549	ICARDA	LR	MR	201	ILWL-398(A)	Lebanon	Wild	S
59	IG-70174	ICARDA	LR	MR	202	ILWL-401	Lebanon	Wild	MR
60	IG-70230	ICARDA	LR	MR	203	ILWL-415	Syria	Wild	S
61	IG-71352	ICARDA	LR	MR	204	ILWL-418	Syria	Wild	MR
62	IG-71630	ICARDA	LR	MR	205	ILWL-428	Spain	Wild	MR
63	IG-71646	ICARDA	LR	MR	206	ILWL-430	Spain	Wild	MR
64	IG-71685	ICARDA	LR	MR	207	ILWL-436	Turkey	Wild	S
65	IG-71710	ICARDA	LR	MR	208	ILWL-437	Turkey	Wild	MR
66	IG-73717	ICARDA	LR	MR	209	ILWL-438	Turkey	Wild	MR
67	IG-73798	ICARDA	LR	MR	210	ILWL-44	Slovenia	Wild	S
68	IG-73802	ICARDA	LR	MR	211	ILWL-447	Turkey	Wild	MR
69	IG-73816	ICARDA	LR	MR	212	ILWL-462	Turkey	Wild	MR
70	IG-73945	ICARDA	LR	MR	213	ILWL-464	Syria	Wild	MR
71	IG-75920	ICARDA	LR	MR	214	ILWL-472	-	Wild	MR
72	IG-9	ICARDA	LR	S	215	ILWL-55(2)	Israel	Wild	MR
73	IG-936	ICARDA	LR	S	216	ILWL-58	Turkey	Wild	MR
74	ILL-10030	ICARDA	GC	MR	217	ILWL-60	Turkey	Wild	MR
75	ILL-10031	ICARDA	GC	MR	218	ILWL-69	Uzbekistan	Wild	MR
76	ILL-10032	ICARDA	GC	MR	219	ILWL-83	Turkey	Wild	MR
77	ILL-10034	ICARDA	GC	MR	220	ILWL-95	Turkey	Wild	MR
78	ILL-10040	ICARDA	GC	MR	221	IPL-406	India	Cult.	MR
79	ILL-10041	ICARDA	GC	MR	222	JL-3	India	Cult.	MR
80	ILL-10043	ICARDA	GC	MR	223	L-404	India	BL	MR
81	ILL-10056	ICARDA	GC	MR	224	L-4076	India	Cult.	MR
82	ILL-10061	ICARDA	GC	MR	225	L-4078	India	BL	MR
83	ILL-10062	ICARDA	GC	MR	226	L-4147	India	Cult.	S
84	ILL-10063	ICARDA	GC	MR	227	L-4578	India	BL	MR
85	ILL-10074	ICARDA	GC	MR	228	L-4590	India	Cult.	S
86	ILL-10075	ICARDA	GC	MR	229	L-4594	India	Cult.	MR
87	ILL-10082	ICARDA	GC	MR	230	L-4602	India	BL	R
88	ILL-10133	ICARDA	GC	MR	231	L-4603	India	BL	MR
89	ILL-10234	ICARDA	GC	MR	232	L-4605	India	BL	MR
90	ILL-10266	ICARDA	GC	MR	233	L-4618	India	BL	MR
91	ILL-10270	ICARDA	GC	MR	234	L-4619	India	BL	MR
92	ILL-1046	ICARDA	GC	MR	235	L-4620	India	BL	MR
93	ILL-10756	ICARDA	GC	MR	236	L-4650	India	BL	MR
94	ILL-10794	ICARDA	GC	R	237	L-4701	India	BL	MR
95	ILL-10795	ICARDA	GC	MR	238	L-5253	India	BL	MR
96	ILL-10804	ICARDA	GC	MR	239	L-7752	India	BL	MR
97	ILL-10805	ICARDA	GC	MR	240	L-7818	India	BL	MR
98	ILL-10806	ICARDA	GC	MR	241	L-7903	India	BL	R
99	ILL-10807	ICARDA	GC	MR	242	L-7905	India	BL	MR
100	ILL-10809	ICARDA	GC	MR	243	L-7920	India	BL	MR
101	ILL-10810	ICARDA	GC	MR	244	LC-270-804	India	BL	MR
102	ILL-10811	ICARDA	GC	MR	245	LC-282-1077	India	BL	MR
103	ILL-10812	ICARDA	GC	MR	246	LC-282-1110	India	BL	MR
104	ILL-10817	ICARDA	GC	MR	247	LC-282-1444	India	BL	MR
105	ILL-10818	ICARDA	GC	MR	248	LC-282-896	India	BL	MR
106	ILL-10819	ICARDA	GC	MR	249	LC-284-116	India	BL	R
107	ILL-10820	ICARDA	GC	MR	250	LC-284-1209	India	BL	MR
108	ILL-10823	ICARDA	GC	MR	251	LC-285-1344	India	BL	MR
109	ILL-10826	ICARDA	GC	MR	252	LC-289-1444	India	BL	MR
110	ILL-10827	ICARDA	GC	MR	253	LC-289-1447	India	BL	MR
111	ILL-10831	ICARDA	GC	MR	254	LC-292-1485	India	BL	MR
112	ILL-10834	ICARDA	GC	MR	255	LC-292-1544	India	BL	MR
113	ILL-10835	ICARDA	GC	MR	256	LC-292-997	India	BL	MR
114	ILL-10836	ICARDA	GC	MR	257	LC-300-1	India	BL	MR
115	ILL-10837	Turkey	GC	MR	258	LC-300-11	India	BL	MR
116	ILL-10848	Bangladesh	GC	MR	259	LC-300-12	India	BL	MR
117	ILL-10857	ICARDA	GC	MR	260	LC-300-13	India	BL	MR
118	ILL-10893	ICARDA	GC	MR	261	LC-300-15	India	BL	MR
119	ILL-10894	ICARDA	GC	MR	262	LC-300-16	India	BL	MR
120	ILL-10897	ICARDA	GC	MR	263	LC-300-2	India	BL	MR
121	ILL-10913	ICARDA	GC	MR	264	LC-300-3	India	BL	MR
122	ILL-10915	ICARDA	GC	MR	265	LC-300-4	India	BL	MR
123	ILL-10917	ICARDA	GC	MR	266	LC-300-6	India	BL	S
124	ILL-10921	ICARDA	GC	MR	267	LC-300-7	India	BL	MR
125	ILL-10922	ICARDA	GC	MR	268	LC-300-8	India	BL	MR
126	ILL-10951	ICARDA	GC	MR	269	LC-300-9	India	BL	MR
127	ILL-10953	ICARDA	GC	MR	270	LC-74-1-51	India	BL	MR
128	ILL-10960	ICARDA	GC	S	271	PAL-3	ICARDA	GC	MR
129	ILL-10961	ICARDA	GC	MR	272	PDL-1	ICARDA	BL	R
130	ILL-10963	ICARDA	GC	MR	273	PDL-2	ICARDA	BL	MR
131	ILL-10964	ICARDA	GC	MR	274	PKVL-1	India	Cult.	MR
132	ILL-10965	ICARDA	GC	MR	275	PL-1	India	Cult.	MR
133	ILL-10967	ICARDA	GC	MR	276	PL-4	India	Cult.	MR
134	ILL-10969	ICARDA	GC	MR	277	PL-406	India	Cult.	MR
135	ILL-10970	ICARDA	GC	MR	278	PL-5	India	Cult.	MR
136	ILL-10972	ICARDA	GC	MR	279	PSL-1	ICARDA	GC	S
137	ILL-1970	Ethiopia	GC	MR	280	PSL-7	ICARDA	GC	R
138	ILL-358	Mexico	GC	MR	281	PSL-9	India	BL	MR
139	ILL-3829	ICARDA	GC	MR	282	SEHORE-74-3	India	Cult.	MR
140	ILL-4605	Argentina	Cult.	MR	283	SKL-259	India	BL	MR
141	ILL-560	Turkey	GC	MR	284	VL-507	India	Cult.	MR
142	ILL-5722	ICARDA	GC	MR	285	WBL-77	India	Cult.	MR
143	ILL-5883	Jordan	GC	MR					

GC = Germplasm collection, BL = Breeding line, Cult. = Cultivar, LR = Landrace, R = resistant, MR = Moderately resistant, S = Sensitive.

### Experimental details

The hydroponic experiments were carried out at National Phytotron facility, Indian Agricultural Research Institute, New Delhi, India. The air temperature in the National Phytotron facility was 23/ 18°C, (±2°C) day/night, and the relative air humidity was approximately 45%. The field experiments were conducted during 2012–13, 2013–14 and 2014–15 growing seasons at Imphal, Manipur, India and Basar, Arunanchal Pradesh, India as these areas are highly affected by Al toxicity in India. The average rainfall (mm) at the two locations in the three consecutive years were 59.0, 28.2, 34.6; 58.9, 60.7 and 52.4, respectively.

### Evaluation of genotypes under hydroponic assay

#### Growth response method

Seeds of 285 genotypes were disinfected and germinated on template stand for hydroponic assay. The seedlings of almost similar length were placed in a plastic container in low-ionic hydroponic medium [KNO_3_ (0.5 mM), Ca(NO_3_)_2_.4H_2_O (0.5 mM), MgSO_4_.7H_2_O (0.2 mM), KH_2_PO_4_ (0.1 mM), KCl (50 μM), H_3_BO_3_ (46 μM), Fe-EDTA (20 μM), MnCl_2_.4H_2_O (2 μM), ZnSO_4_.7H_2_O (1 μM), CuSO_4_.5H_2_O (0.3 μM) and NaMoO_4_.2H_2_O (0.5 μM)] [24]. The pH of nutrient solution was maintained at 4.5 for both 74 μM and 148 μM Al concentrations using 1 M HCl or 1 M KOH. The solutions were replaced daily to minimize changes in Al concentrations and pH. After one week, the lengths of root and shoot of each seedling was measured. The seedlings were evaluated in a completely randomized design with three replications.

#### Root re-growth (RRG) after haematoxylin staining method

Aluminium resistance for 285 genotypes was evaluated in a nutrient solution by testing -RRG after haematoxylin staining of root apices following the protocol of Polle *et al*. with partial modifications [25]. Seeds were disinfected with 0.1% HgCl_2_ for 2–3 min, rinsed thoroughly with distilled water, then transferred to filter paper and placed in the growth chamber for germination. After 6 days, seedlings were transferred to plastic containers containing nutrient solution (4.0 mM CaCl_2_, 6.5 mM KNO_3_, 2.5 mM MgCl_2_, 0.1 mM (NH_4_)_2_SO_4_, 0.4 mM NH_4_NO_3_). Seedlings were kept in above nutrient solution for 2 days under continuous light and aeration. The seedlings were then grown for 24 h on the fresh nutrient solution containing 74 and 148 μM Al concentrations. The roots of seedlings were then placed in aerated distilled water, washed for 30 min to remove Al from the root surface and then stained. The staining solution consisted of 2 g/L of haematoxylin and 0.02 g/L KIO_3_, which was prepared in distilled water 24 h before the assay. The roots of seedlings were immersed in the haematoxylin stain for 15–30 min after which the seedlings were transferred through three washes in deionised water for 30 min to remove excess stain. Each seedling was visually scored on the pattern of staining of the primary root tip as suggested by Polle *et al*. [25]. The seedlings were then returned to the nutrient solution without Al and kept for 48 h. The response of each genotype was determined as re-growth of the primary root after staining. Genotypes with mean primary RRG <0.5 cm were classified as Al sensitive, while those with mean primary RRG significantly >1.0 cm were considered resistant. Seedlings showing intermediate RRG (0.50–1.00 cm) were rated as moderately resistant. The experiment was conducted in a completely randomized design with two replications. Six seedlings per replications were evaluated for determination of Al resistance.

### Molecular assortment analysis

#### DNA extraction

Genomic DNA was isolated from the fresh leaf samples of all the 285 genotypes (10 plants per individual) using conventional CTAB method described by Doyle and Doyle [26]. To check the quantity, it was compared with lambda uncut DNA on 1% ultra high resolution agarose gel and quality was determined by using spectrophotometer. Standard working concentration of 50 ng/μl of DNA sample was used.

#### SSR marker analysis

Molecular diversity analysis was performed using a total of 495 SSR primers reported by Hamwieh *et al*., Kaur *et al*. and Jain *et al*. [27, 28, 29]. To shortlist the number of markers, these markers were initially screened between the most resistant (L-7903, L-4602) and sensitive (BM-4, L-4147) genotypes which were identified in morpho-physiological analysis. Seventy three SSR primers which exhibited polymorphism between these contrasting genotypes were then used for analysis of genetic diversity among all the 285 genotypes. The primers were synthesized by Microgen, South Korea and IDT, USA. Polymerase chain reaction was performed in 10 μl reaction mixture comprising of 1X PCR buffer, 0.5 U *Taq* DNA polymerase, 1 mM dNTP, 0.5 μM of forward and reverse primers each (Microgen, South Korea and IDT, USA) and 50 ng/μl of genomic DNA in a thermocycler (Agilent Technologies, USA). The PCR protocol comprised of initial denaturation step of 94°C for 3 min followed by 40 cycles of 94°C for 1 min, annealing at 55°C for 30 sec, elongation at 72°C for 30 sec with final extension at 72°C for 10 min. The amplified products were resolved on 3% ultra high resolution agarose gels and documented using Vilber Lourmat Gel Documentation System (Model Bio-print ST4, France).

#### Genetic diversity analysis

The genetic profile of 285 lentil genotypes was scored on the basis of difference in allele size using 46 SSR markers. The major allele frequency, PIC and genetic distance based clustering was performed with Unweighted Pair Group Method for Arithmetic average (UPGMA) tree using Power Marker v3.25 software [30] and the dendrogram was constructed following bootstrap analysis with 1000 permutations for all the genotypes using MEGA 4.0 software [31]. The population structure for 285 lentil genotypes comprising both wilds and cultigens was inferred using Structure 2.3.4 software [32]. The structure outputs were visualized using Structure Harvester from which Evanno plots were constructed [33]. An assumed admixed model with independent allele frequency and a uniform prior probability of the number of populations, *K* was used in the Structure. All the runs were conducted for *K* = 1 to 15 with 1,00,000 Monte Carlo Markov Chain (MCMC) replicates after a burn-in of 10,000 replicates. For each value of *K*, 7 independent runs were done to generate an estimate of the true number of sub-populations [32]. The relation between genetic similarity identified by SSR markers and taxonomic distance measured by mean genetic distance and RRG after haematoxylin staining were analysed using Jaccard’s Similarity Index and average taxonomic distance was calculated by NTSYS-pc v2.1 software [34]. Duncan’s Multiple Range Test (DMRT) (*P* = 0.05) was used to evaluate differences among clusters for significance by using SPSS v16.0 software.

### Physiological assessment of contrasting genotypes

Contrasting genotypes based on morphological evaluation which included resistant breeding lines (L-7903, L-4602), wild accession (ILWL-185) and sensitive -cultivars (BM-4, L-4147), sensitive wild accession (ILWL-436) were further evaluated for Al content, callose accumulation, detection of Al accumulation through morin, detection of Hydrogen peroxide (H_2_O_2_) level, determination of lipid peroxidation, antioxidant and organic acid under 0, 74 μM and 148 μM Al treatment.

#### Determination of Al contents in roots and shoots

For determination of Al content, dry samples of roots and shoots were ground and dissolved in a di-acid mixture (nitric acid and perchloric acid) in 3:1 ratio (v/v). Al contents were quantified using Perkin-Elmer Atomic Absorption Spectrophotometer (Model 5000, Perkin-Elmer, Shelton, CT-USA). Six roots and shoots from each treatment were analysed for determination of Al contents. The amount of Al was expressed in mg/g on dry weight basis.

#### Detection of callose accumulation

Fifteen roots of the seedlings were collected from each treatment after 48 h exposure to Al stress and were transferred to fixative FAA (10% formaldehyde, 5% glacial acetic acid and 10% ethanol) for 1 h and then root tips (first centimetre) were excised and washed in deionised water. Lowest 1 cm segment of root tips were stained for 10 min in a solution containing 0.1% water soluble aniline blue in 50 mM Glycine-NaOH buffer at pH 9.5 [35]. After staining, callose production was visualized under fluorescence microscope (Zeiss, AXIOSKOP 2). Seedlings were then visually scored as suggested by Singh *et al*. [36].

#### Detection of aluminium accumulation by morin staining technique

Localization of Al in roots was visualized by staining with morin dye, which forms highly specific complexes with Al at low pH. The seedlings were subjected to Al stress for 48 h under conditions of 0, 74 and 148 μM Al in nutrient solution (pH 4.5). The roots of Al stressed and non-stressed plants were washed in buffer (5 mM NH_4_OAc, pH 5.0±0.01) for 10 min and stained with 100 μM morin in the same buffer for 30 min and finally washed with NH_4_OAc buffer for 10 min [37]. Morin fluorescence was visualized using fluorescence microscope.

#### Detection of H_2_O_2_ level

For detection of H_2_O_2_ level, fifteen Al treated root tips were collected from each treatment, excised and placed into a solution containing 200 mM CaCl_2_ (pH 4.4) and 10 mM 2’,7’-dichlorofluorescein diacetate (DCF-DA) for 15 min. The DCF-DA fluorescence was then detected under a fluorescence microscope.

#### Determination of lipid peroxidation

For determination of lipid peroxidation, Thiobarbituric acid reactive substances (TBARS) were estimated following the method of Heath and Packer and expressed in malondialdehyde (MDA) concentration [38]. Fresh root and shoot samples (0.1 g) were homogenized in liquid nitrogen with 2 ml of 0.1% (w/v) trichloro acetic acid (TCA) and were centrifuged. The reaction mixtures [0.1 ml supernatant and 40 ml of thiobutyric acid (0.5% in 20% TCA)] were then heated at 95°C for 30 min and the reactions were stopped by cooling them in ice bath and were again centrifuged. Supernatants were collected and absorbance was measured at 532 and 600 nm and non-specific absorbance measured at 600 nm was subtracted to determine MDA content using the extinction coefficient of 155 mM^-1^ cm^-1^ and was expressed as μmol g^-1^ fresh weight.

#### Determination of antioxidant enzymes

Six root and shoot samples from each treatment were collected for determination of antioxidant enzyme activities after 48 h exposure to Al stress. SOD, APX, GPX and CAT enzymes were extracted by grinding 1.0 g fresh shoot and root samples in 10 ml extraction buffer (0.1 M phosphate buffer, pH 7.5, containing 0.5 mM EDTA in case of SOD, CAT and GPX; 1 mM ascorbic acid for APX). The extract was passed through four layers of cheese cloth and centrifuged. Thereafter supernatant was collected and used as enzyme extract. Assay for each enzyme was conducted following the methods outlined below:

1. **CAT activity** was determined following the procedure of Aebi [39]. Three milliliter reaction mixture was prepared using phosphate buffer (50 mM, pH 7.0), 15 mM H_2_O_2_ and 0.1 ml enzyme extract. Absorbance was recorded at 240 nm using UV-Visible spectrophotometer (Specord Bio 200 Analytik Jena, Germany).
2. **SOD activity** was determined by recording the enzyme induced reduction in absorbance of formazone made by reaction of p-nitroblue tetrazolium chloride (NBT) and superoxide radicals following the procedure of Dhindsa *et al*. [40]. Reaction was initiated by adding 2 μM riboflavin in a reaction mixture containing potassium phosphate buffer (50 mM, pH 7.8), 13.33 mM methionine, 75 μM NBT, 0.1 mM EDTA, 50 mM sodium carbonate and 0.1 ml enzyme extract. The test tubes containing reaction mixture were exposed to light under 15 W fluorescent lamps for 15 min. A similar set of reaction mixture (without enzyme) was used as control and a non-irradiated complete reaction mixture served as blank. The absorbance was recorded at 560 nm using UV-Visible spectrophotometer (Specord Bio 200 Analytik Jena, Germany). One unit of SOD activity was expressed as the amount of enzyme that inhibited 50% NBT photoreduction.
3. **APX activity** was determined by recording the reduction in ascorbate following the procedure of Nakano and Asada [41]. The reaction mixture containing potassium phosphate buffer (50 mM, pH 7.0), 0.5 mM ascorbic acid, 0.1 mM EDTA, 0.1 mM H_2_O_2_ and 0.1 ml enzyme was used for assay and reaction was started with the addition of hydrogen peroxide. Absorbance was measured at 290 nm using UV-Visible spectrophotometer (Specord Bio 200 Analytik Jena, Germany).
4. **GPX activity** was assayed by monitoring the formation of tetraguaiacol from guaiacol at 470 nm in the presence of H_2_O_2_ [42, 43]. The reaction mixture containing 16 mM guaiacol, 2 mM H_2_O_2_, phosphate buffer (50 mM, pH 7) and 20 μl enzyme extract was used for assay. Absorbance was recorded at 470 nm using UV-Visible spectrophotometer. The unit activity of all the above enzymes was expressed as g^-1^ min^-1^ fresh weight.

#### Effect of Al concentration and treatment time for determination of organic acid

Root exudates were collected following the method of Zhao *et al*. [44] with modifications. Seven days old seedling (twelve seedlings) with almost similar length were placed into separate plastic container (1 L volume) and exposed to 0.5 mM CaCl_2_ solution with 0, 74 and 148 μM Al (AlCl_3_.6H_2_O, pH 4.5) to collect the root exudates (15 ml from each container) under growth chamber. Root exudates were collected at 30 min, 1, 3 and 6 h after the start of Al treatments. Growth chamber was programmed at 21–17°C day/night temperature and 10 h day/14 h night cycle; with light intensity maintained at 550 μmol m^-2^ s^-1^ and percent relative humidity was 45±2%.

Collected root exudates (15 ml) were passed through a cation-exchange resin column (16 mm · 14 mm) filled with 5 g of Amberlite IR-120 B resin (H+ form) then concentrating it to 1.5 ml with the help of speed vacuum centrifuge (Eppendof, Germany) and purified by using nitrocellulose syringe filter with mesh of 45 mm (Millipore, USA). Purified extract (100 μL) was acidified to approx. 4.0 pH, injected into High performance liquid chromatography (HPLC) [Agilent Hi-Plex Ligand Exchange Column, Hi-Plex H, 7.7 x 300 mm, 8 μm (p/n PL 1170–6830 column), Germany] and run isocratically at 70°C using 5 mM H_2_SO_4_ as the mobile phase at a flow rate 0.6 ml min^-1^. The specific organic acids were quantified based on their refractive index against standards and expressed in nmol h^-1^ g^-1^ fresh weight.

### Evaluation of selected genotypes in acidic conditions

The experiments were carried out at Andro Farm, Central Agricultural University, Imphal, Manipur, India and Krishi Vigyan Kendra (KVK), Indian Council of Agricultural Research (ICAR) Research Complex for North Eastern Hill (NEH) region, Basar, Arunachal Pradesh, India during 2012–13, 2013–14 and 2014–15 growing seasons under acidic conditions. Soil samples were collected from 0–20 cm soil depth and were analysed for organic carbon [45], exchangeable Al [46], available N, P [47], K, Ca and Mg [48]. The soil of the experimental fields at Imphal, Manipur, India and Basar, Arunachal Pradesh, India (1:2::soil:water) had pH 4.8 and 5.1; EC 0.27 dsm^-1^ and 0.31 dsm^-1^, organic content of 0.56% and 0.71%, respectively. The availability of N, P, K, Ca and Mg (kg/ha) were 259 and 281; 16.8 and 18.5; 119 and 126.4; 522 and 179; 249 and 261 at the two locations, respectively. The exchangeable Al in the soils at Imphal and Basar, which were extracted with 1 M KCl was 1.28 and 1.17 meq/100 g, respectively. Further, soil cation-exchange capacity for the two locations, Imphal and Basar were 12 and 13 meq/100 g. Therefore, aluminium saturation percentage of soil at Imphal and Basar were calculated to as 9.15 and 9.0, respectively. Field experiments were conducted in a randomized block design with three replications. Each experimental plot had 6 rows of 5 m length with inter and intra row spacing of 20 cm and 2.5 cm, respectively. The normal package and practices were followed.

## Results

### Phenotyping for Al resistance based on morphological traits

The effect of Al toxicity was more prominent on roots as compared to shoots (data not shown). Highly significant differences were found in the reduction of root and shoot lengths and RRG after haematoxylin staining. However, there was no significant difference in root colour intensity among genotypes after staining. The resistant cultigens showed 13.20–24.38% reduction in root length and 6.96–14.48% reduction in shoot length. However, sensitive genotypes showed 41.50–75.10% reduction in root length and 17.53–54.65% reduction in shoot length when treated with Al stress for 8 d. Reduction in root and shoot lengths in moderately resistant cultigens were from 16.17–49.07% and 4.67–42.49%, respectively. Resistant wild accessions showed 12.07–18.09% reduction in root length, 8.45–9.17% reduction in shoot length and sensitive wild accessions showed 70.54–50.33% reduction in root length and 45.27–27.12% reduction in shoot length. RRG after haematoxylin staining in resistant cultigens was from 1.20–1.60 cm, while in sensitive cultigens it ranged from 0–0.47 cm. Moderately resistant cultigens showed 0.43–1.00 RRG. RRG in resistant wild accessions was 0.80–1.87 cm and in sensitive wilds it ranged from 0.37–0.47 cm. Moderately resistant wild accessions showed range of 0.70–0.97 cm RRG.

### Molecular assortment of *Lens* species

#### SSR molecular marker analysis

A set of 495 primers were pre-screened in couple of Al resistant (L-7903 and L-4602) and sensitive (BM-4 and L-4147) genotypes, of which 73 SSR primers which exhibited polymorphism were selected for genetic diversity analysis among 285 genotypes (Table 2). Out of 73 SSR markers 46 SSR markers generated polymorphic bands among the genotypes with comparatively higher PIC. Remaining 27 primers were not used for further analysis as their PIC values were very low. A total of 193 alleles were identified with an average of 4.11 alleles per locus. The number of alleles per locus ranged from 2 (PBA_LC_278, PBA_LC_327, PBA_LC_376, PBA_LC_404, PLC_60, PBA_LC_1308, PBA_LC_1241 and PLC_88) to 11 (LC_03). The gene diversity and PIC values varied between 0.148–0.775 and 0.140–0.739, with an average of 0.484 and 0.428, respectively. The primer which showed highest gene diversity and PIC value was LC_02 while the lowest gene diversity and PIC value was observed for the primer PBA_LC_949. Heterozygosity in all the genotypes ranged from 0 to 0.575 with a mean value of 0.106 and the highest heterozygosity was observed in primer LC_01 (Table 2). The major allele frequency varied between 0.300 (PBA_LC_333) to 0.921 (PBA_LC_949) with a mean value of 0.637. A representative profile of 48 cultigens (out of 210) and 48 wilds (out of 75) with SSR marker PLC_100 and PBA_LC_1551, respectively are represented in S1 and S2 Figs.

**Table 2:** Allelic variations and PIC values for SSR markers identified in 285 lentil genotypes.

Marker	Allele No.	Major Allele Frequency	Gene Diversity	Heterozygosity	PIC
PLC-35	4	0.787	0.365	0.150	0.346
PLC-05	4	0.601	0.581	0.207	0.538
PLC-30	3	0.726	0.404	0.028	0.331
PLC-39	3	0.900	0.180	0.004	0.165
PLC-81	3	0.772	0.374	0.107	0.339
PLC-91	3	0.600	0.526	0.077	0.443
PLC-100	6	0.392	0.703	0.120	0.648
PLC-104	5	0.439	0.689	0.051	0.637
LC-01	6	0.504	0.594	0.575	0.514
LC-02	9	0.305	0.775	0.358	0.739
LC-16	4	0.867	0.235	0.142	0.216
PBA-LC-221	5	0.690	0.477	0.077	0.432
PBA-LC-222	3	0.668	0.497	0.059	0.444
PBA-LC-368	4	0.578	0.554	0.060	0.479
PBA-LC-377	4	0.570	0.578	0.015	0.512
PBA-LC-379	4	0.794	0.350	0.174	0.325
PLC-51	3	0.553	0.524	0.000	0.419
PBA-LC-376	2	0.822	0.293	0.000	0.250
PBA-LC-117	4	0.593	0.565	0.157	0.506
PBA-LC-118	5	0.623	0.490	0.019	0.395
PBA-LC-404	2	0.783	0.340	0.000	0.282
PBA-LC-1241	2	0.642	0.460	0.000	0.354
PBA-LC-652	3	0.679	0.485	0.129	0.433
PBA-LC-1247	3	0.751	0.393	0.000	0.344
PLC-60	2	0.754	0.371	0.000	0.302
PLC-88	2	0.618	0.472	0.050	0.361
PBA-LC-373	3	0.489	0.543	0.058	0.438
PBA-LC-949	3	0.921	0.148	0.000	0.140
PBA-LC-1351	3	0.639	0.503	0.189	0.430
PBA-LC-1526	8	0.333	0.765	0.158	0.728
PBA-LC-1551	3	0.828	0.290	0.040	0.256
PBA-LC-1375	3	0.643	0.469	0.031	0.373
PBA-LC-1401	7	0.438	0.697	0.455	0.648
PBA-LC-1698	3	0.485	0.546	0.053	0.442
PBA-LC-278	2	0.904	0.174	0.192	0.159
PBA-LC-327	2	0.898	0.183	0.000	0.167
PBA-LC-216	3	0.623	0.516	0.007	0.440
PBA-LC-1363	4	0.527	0.631	0.075	0.576
PBA-LC-1308	2	0.743	0.382	0.000	0.309
PBA-LC-383	3	0.579	0.539	0.087	0.454
PBA-LC-418	4	0.849	0.271	0.035	0.258
LC-03	11	0.385	0.719	0.308	0.677
PBA-LC-1530	5	0.571	0.603	0.019	0.554
PBA-LC-1554	7	0.481	0.711	0.150	0.681
PBA-LC-333	8	0.300	0.760	0.291	0.720
PLC-46	7	0.654	0.536	0.258	0.504
Mean	4.109	0.637	0.484	0.108	0.428

#### Cluster analysis using molecular markers and morphological traits

**Fig 1:**
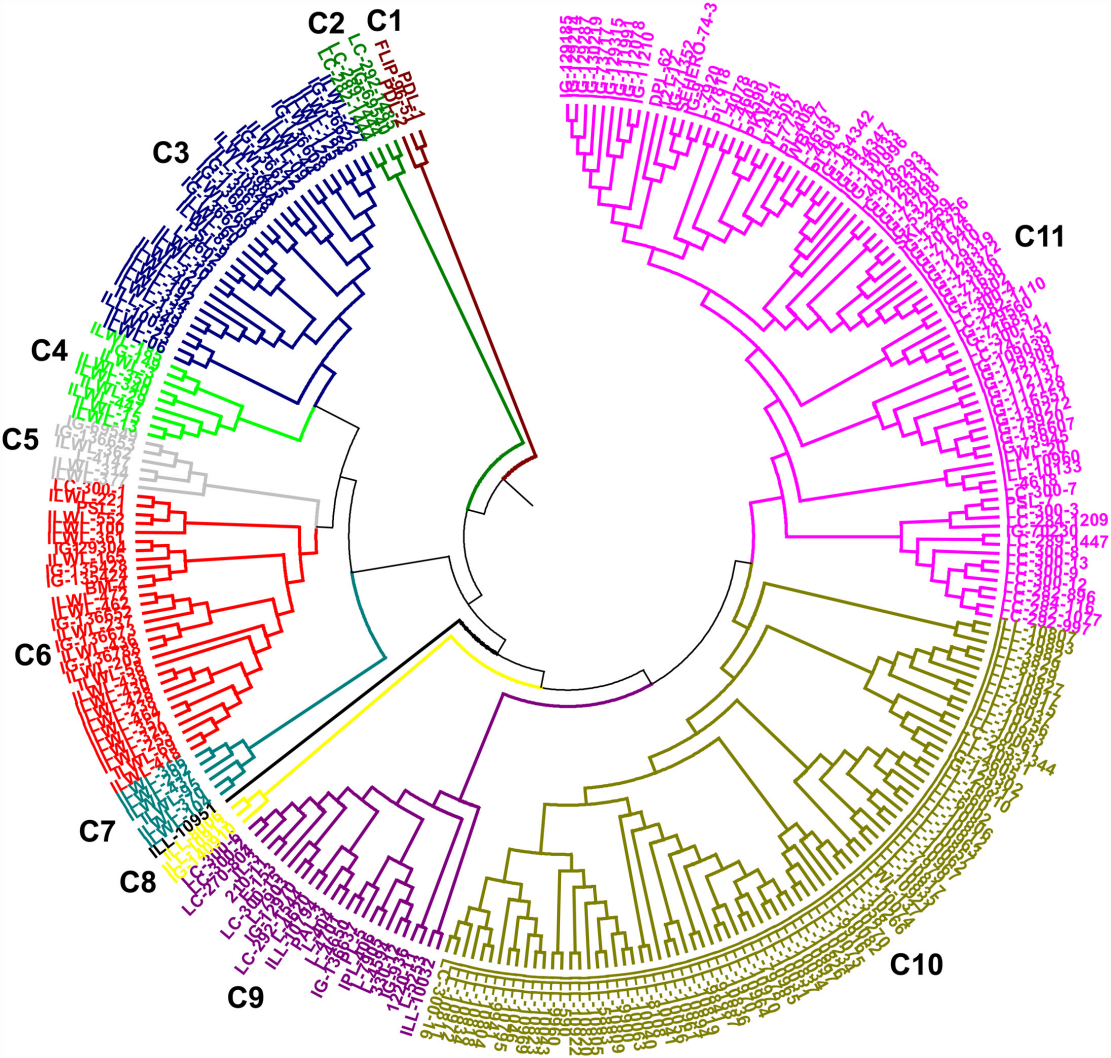
Unweighted Pair Group Method for Arithmetic average (UPGMA) tree based on dissimilarity index of 46 simple sequence repeat (SSR) markers for 285 lentil genotypes.

The genetic relationships among lentil genotypes are presented in SSR based UPGMA tree (Fig 1). All the genotypes are grouped into 11 clusters. The genotype ILL-10951 was distinct and not included into any cluster. Resistant cultigens were mainly grouped into cluster C9 while sensitive ones fall into C5 and C6 clusters. Clusters 3 to 7 grouped wild accessions, where resistant wild accessions were mainly included into C4 and sensitive wild accessions were grouped into C3. Clusters C10 and C11 consisted of 80 and 85 genotypes, respectively, where C10 grouped the “ILL” series of genotypes mainly. The mean genetic distance of the clusters ranged from 0.48 to 0.65 with an average of 0.56. Cluster C1 showed highest genetic distance (0.65) followed by C7 (0.64); C2 (0.61); C8 (0.58); C5 (0.56); C4 (0.55); C3, C6 and C9 (0.54); C10 (0.51) and C11 (0.48) (Table 3).

**Table 3:** Cluster means of reductions in root length and shoot length, root re-growth (RRG) and mean genetic distance (MGD) under Al stress conditions among the clusters.

Clusters	Genotypes	Root length	Shoot length	RRG	MGD
Cluster 1	3	25.94^a^	10.27^abc^	0.91^bc^	0.65^g^
Cluster 2	4	37.60^cd^	18.84^ab^	0.78^ab^	0.61^ef^
Cluster 3	32	40.15^bcd^	24.08^bc^	0.76^a^	0.54^bcd^
Cluster 4	9	27.97^ab^	14.76^a^	1.28^c^	0.55^cd^
Cluster 5	6	45.78^cd^	23.30^abc^	0.72^a^	0.56^cd^
Cluster 6	30	42.87^cd^	25.64^c^	0.80^a^	0.54^bc^
Cluster 7	6	43.62^d^	24.42^abc^	0.81^ab^	0.64^fg^
Cluster 8	3	32.18^ab^	19.62^abc^	0.83^ab^	0.58^de^
Cluster 9	26	35.53^abc^	18.01^abc^	0.74^a^	0.54^bcd^
Cluster 10	80	35.50^abcd^	17.83^abc^	0.79^a^	0.51^ab^
Cluster 11	85	39.81^bcd^	20.82^abc^	0.72^a^	0.48^a^

Values within each column that do not share common letter are significantly different by Duncan’s post- hoc test at P≤0.05.

**Fig 2:**
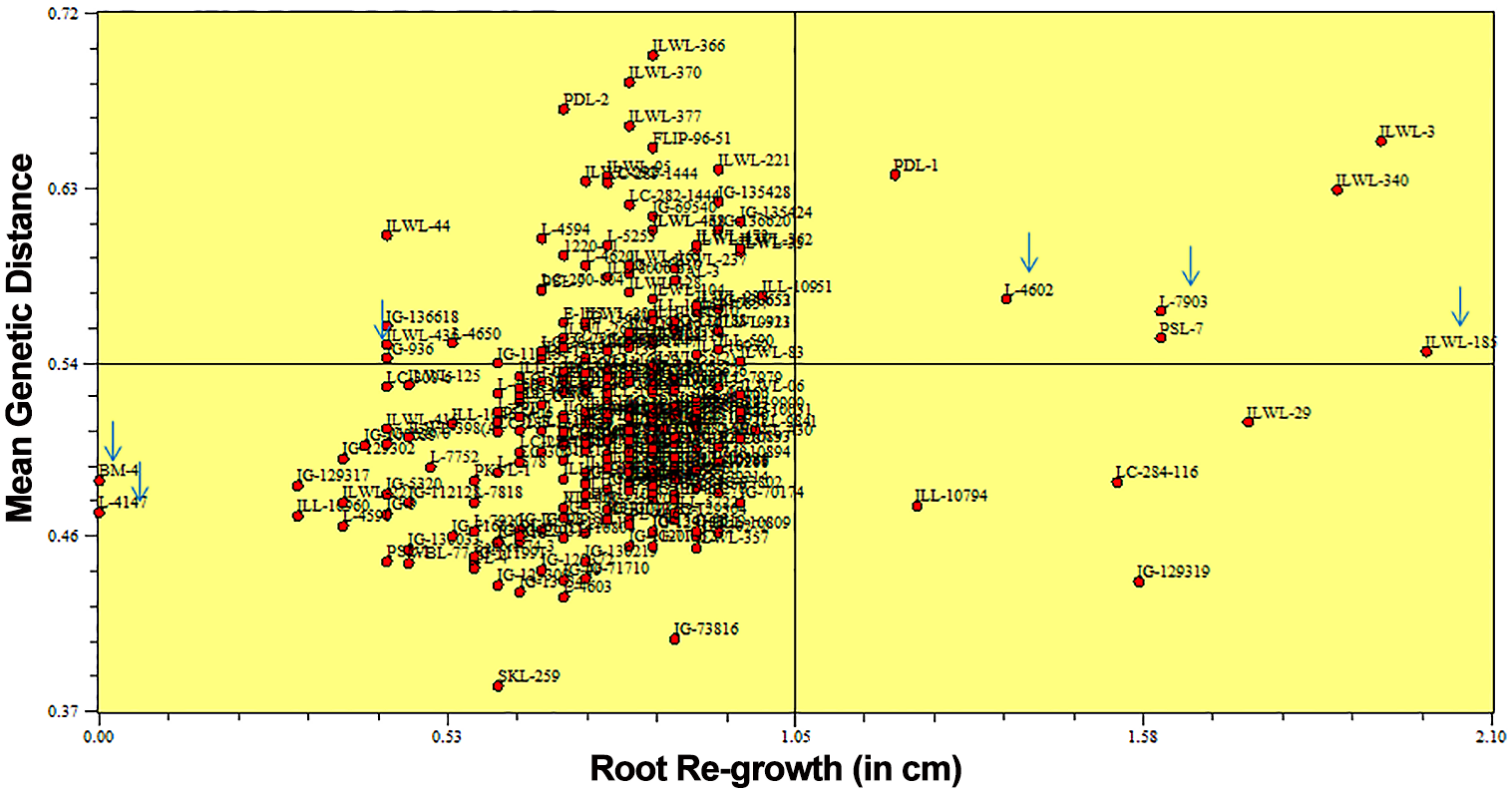
Correlation between genetic similarity measured by mean genetic distance (MGD) and root re-growth (RRG) (cm) in 285 lentil genotypes. Six contrasting genotypes selected for further evaluation for physiological parameters are marked.

The average reduction per cent of root length, shoot length, RRG after haematoxylin staining and mean genetic distance under aluminium stress were calculated among the clusters categorized by SSR markers of all the genotypes. Among the SSR clusters there was wide variation in values for most of the characters analyzed. Significant (*P* = 0.05) differences for all the characters were observed among the clusters. These parameters differed significantly for the genotypes of cluster C4 and C1 as compared to those of other clusters. The lowest reduction of root and shoot length and maximum RRG were observed in the resistant genotypes of clusters C4 and C1 compared to those of other clusters (Table 3). These differences in the growth parameters and physiological traits may be due to strong aluminium resistance among genotypes of clusters C4 and C1. The two selected resistant breeding lines *i*.*e*. L-7903 and L-4602 fall into C9 and C3 clusters, respectively, while the selected sensitive cultivars namely; BM-4 and L-4147 fall into clusters C6 and C5, respectively. Selected wild resistant accession *i*.*e*. ILWL-185 and sensitive wild accession *i*.*e*. ILWL-436 fall into C4 and C6 clusters, respectively. Further, resistant and sensitive genotypes were also separated when correlation between genetic similarity index and taxonomic distance for RRG after haematoxylin staining was evaluated using Jaccard similarity index (Fig 2). Highly significant correlation (r = −0.38; P = 0.005) was observed between the matrices of Jaccard genetic similarity based on SSR and taxonomic distance based on RRG.

#### Population Structure analysis

**Fig 3:**
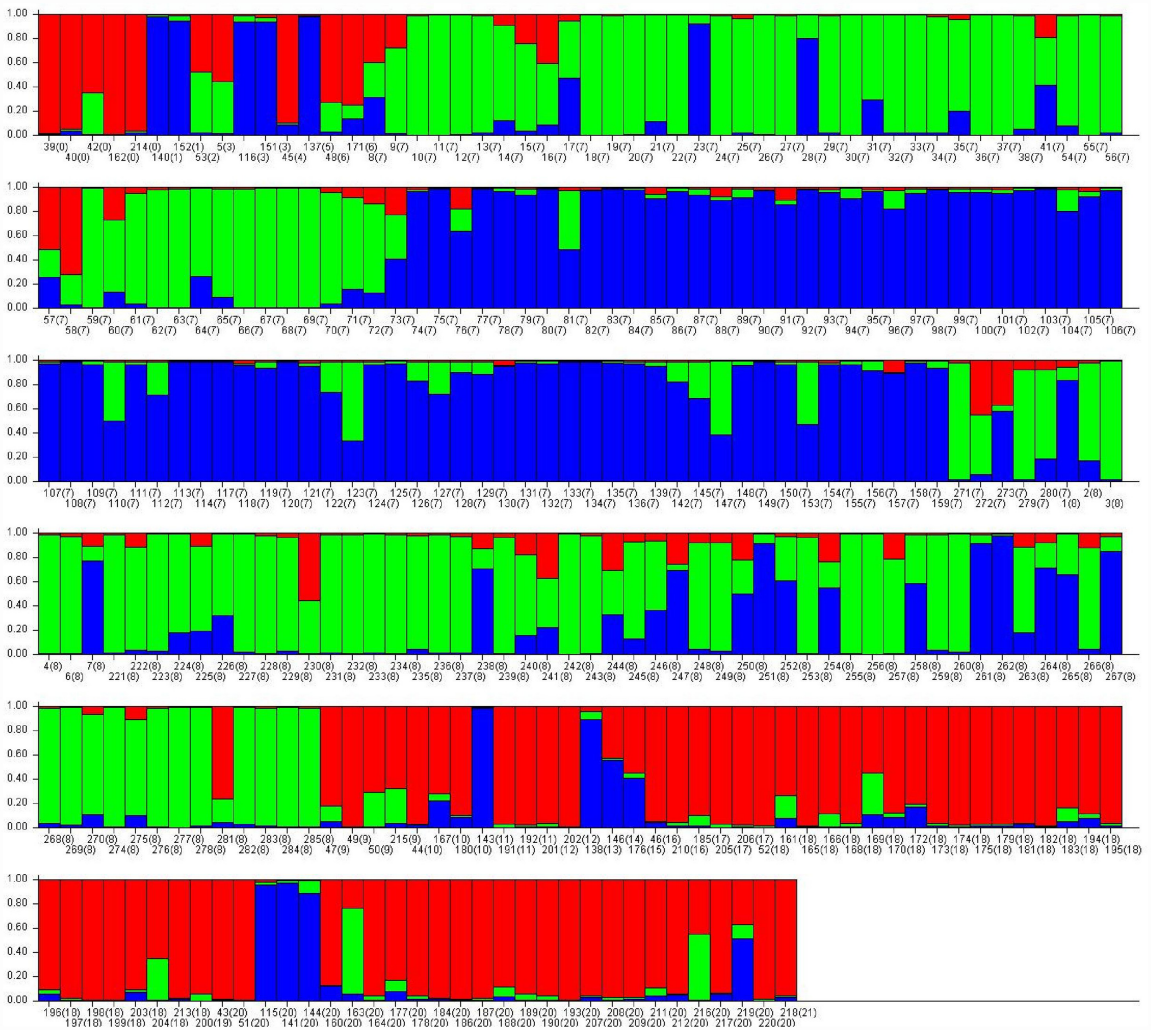
Model based population structure plot with K = 3, using Structure with 46 SSR markers. Colour codes: red = population I (wild accessions), green = population II (cultigens) and blue = population III (‘ILL’ series of cultigens).

The population structure of the 285 lentil genotypes was estimated using STRUCTURE v2.3.3 software based on 46 SSR markers. The optimum *K* value was determined by using Structure Harvester, where the highest peak was observed at delta K = 3 (S3 Fig). The number of subpopulations (*K*) was identified based on maximum likelihood and delta K (dK) values, with accessions falling into three subgroups (Fig 3). Each segment number corresponds to the genotype in Table 1 and the number in brackets represents the population ID assigned on the basis of origin *viz*. Argentina (1), Azerbaijan (2), Bangladesh (3), Croatia (4), Ethiopia (5), France (6), ICARDA (7), India (8), Israel (9), Italy (10), Jordan (11), Lebanon (12), Mexico (13), Nepal (14), Palestine (15), Slovenia (16), Spain (17), Syria (18), Tajikistan (19), Turkey (20) and Uzbekistan (21). Using a membership probability threshold of 0.80, 50 genotypes were assigned to subgroup (SG) 1, 62 genotypes to SG 2 and 48 to SG 3. One hundred twenty five genotypes were retained in the admixed group (AD). The relationship between subgroups derived from STRUCTURE explained that SG 1 comprised of wild types and SG 2 and SG 3 consisted of cultivars, where SG 3 comprised of ‘ILL’ series of cultivars which are mostly germplasm collections. This indicated that the population structure was in accordance with clustering of lentil genotypes in UPGMA tree.

### Physiological dissection of component mechanisms of Al resistance

#### Al accumulation in roots and shoots

**Fig 4:**
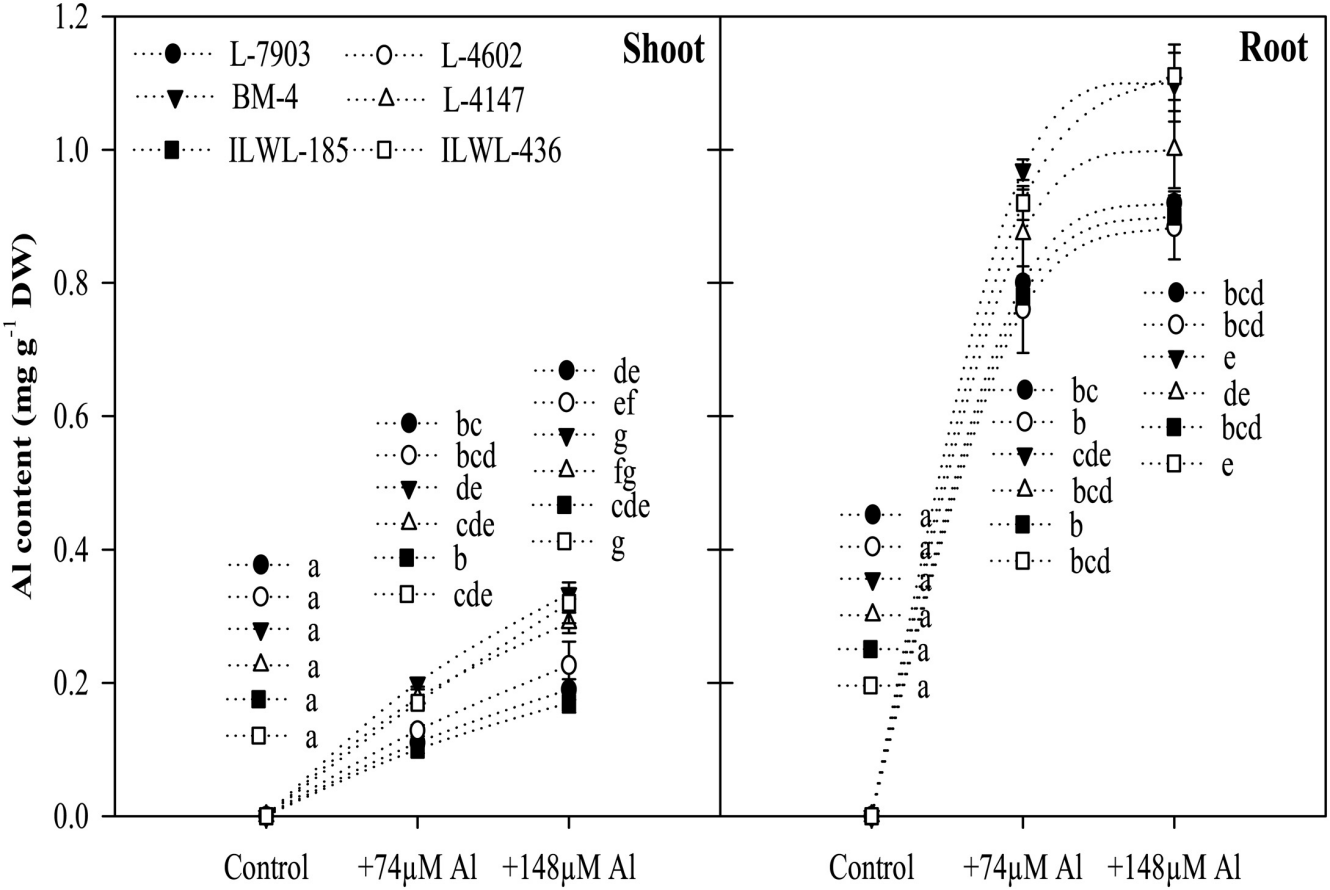
Al contents in root and shoot of lentil: resistant breeding lines (L-7903, L-4602), sensitive cultivars (BM-4, L-4147) and resistant wild accession (ILWL-185), sensitive wild accession (ILWL-436), after exposure to two Al concentrations (0, 74 and 148 μM) for 48 h duration. Mean values with same small letters for each part of the plant do not statistically differ by the Tukey test at P≤0.05.

The Al contents in roots and shoots increased with increase in concentration of Al in the nutrient solution and it was found higher in roots as compared to shoots. Significantly (P<0.05) higher Al content was detected in roots of sensitive cultivars (BM-4, L-4147) than in resistant breeding lines (L-7903, L-4602). In wild type also, resistant accession, ILWL-185, showed lower accumulation of Al as compared to sensitive wild, ILWL-436 (Fig 4).

#### Localization of Al in root tissues

**Fig 5:**
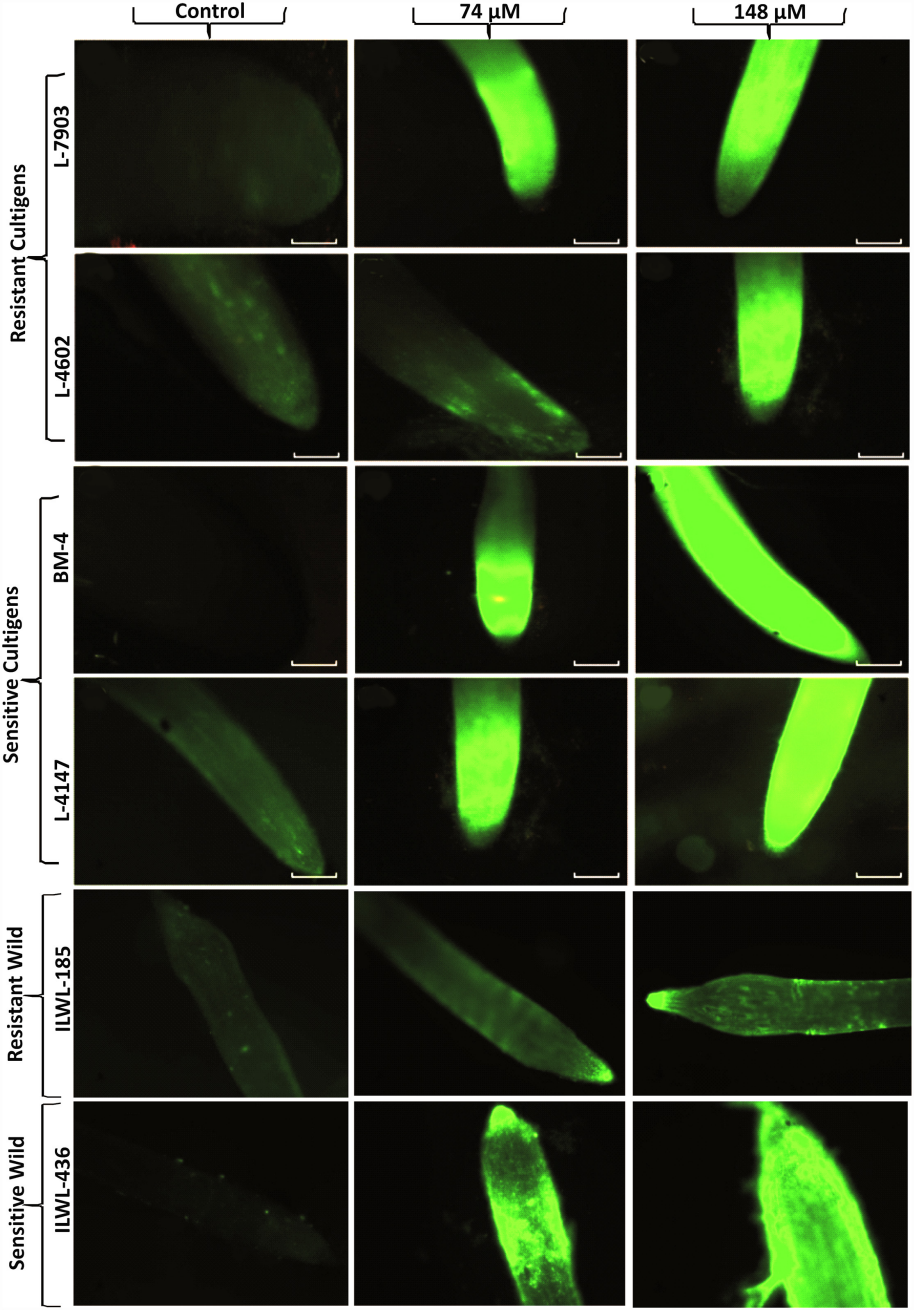
Staining of root tips of Al resistant and sensitive lentil genotypes showing morin staining fluorescence images under control, 74μM and 148 μM Al concentrations. Bar in each figure represents 1 mm.

Morin dye staining technique was used to detect the presence of Al in plant tissues after short-term exposure to Al [25, 37]. This dye binds strongly with Al, where it gives fluorescence under fluorescent microscope (Fig 5). Through morin staining, wild resistant accession, ILWL-185 showed less fluorescence (Al concentration) in its root tip than the resistant cultigens, whereas sensitive wild, ILWL-436 showed similar fluorescence to that of sensitive cultigens.

#### Effects of Al stress on accumulation of callose in roots

**Fig 6:**
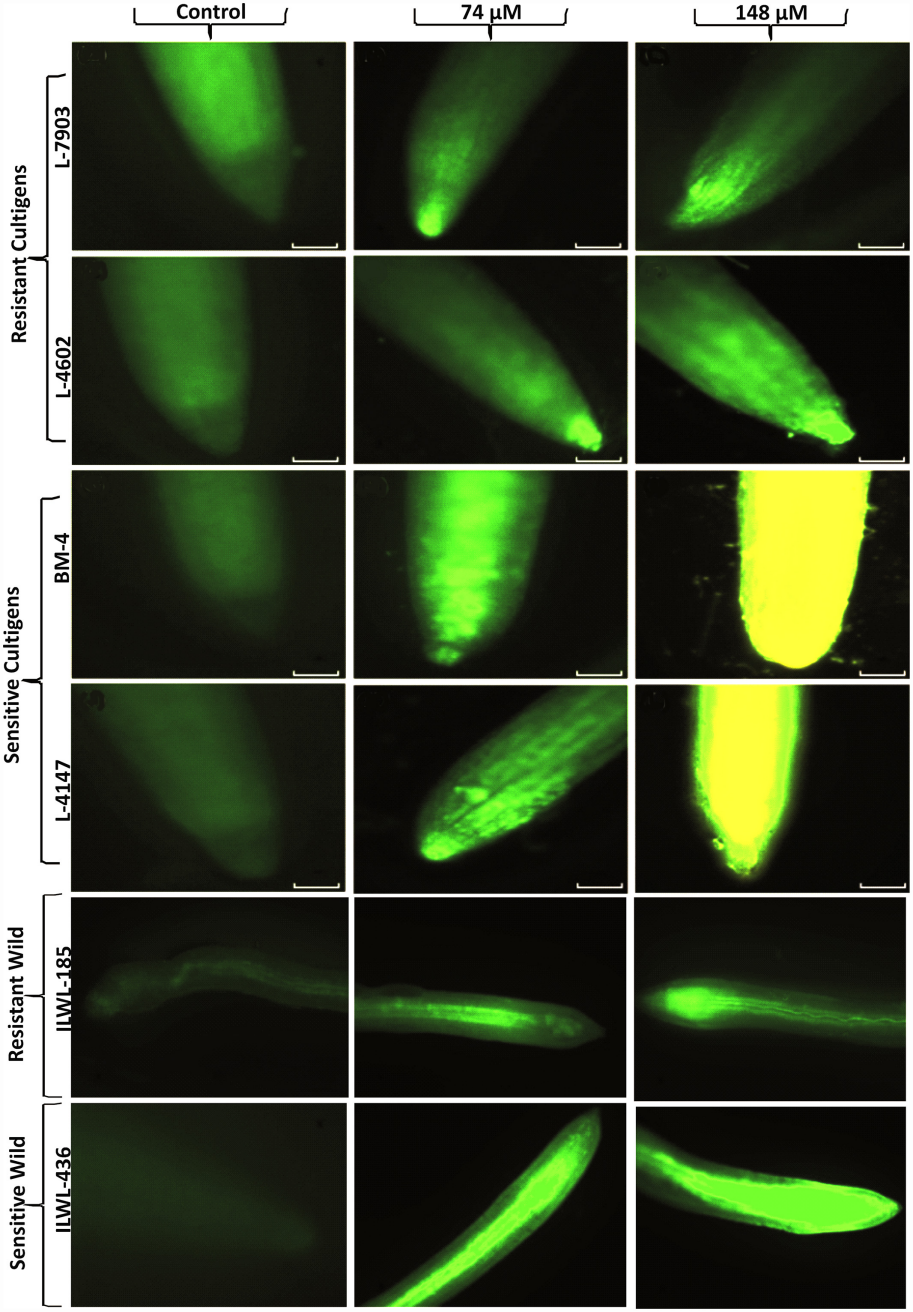
Root tip of lentil seedling showing callose accumulation under control, 74μM and 148 μM Al concentrations. Bar in each figure represents 1 mm.

Roots showed more callose deposition with increasing A1 concentrations from 0 to 148 μM and deposition was consistent with the level of Al-induced root growth inhibition. Callose staining was much less in resistant–breeding lines (L-7903 and L-4602) in comparison to sensitive cultivars (BM-4 and L-4147). Wild resistant accession (ILWL-185) showed less callose accumulation as compared to cultigens under both the treatment conditions *viz*. 74 and 148 μM Al concentrations, whereas sensitive wild, ILWL-436 accumulated more callose (Fig 6).

#### Effects of Al stress on H_2_O_2_ production

Hydrogen peroxide production in roots was visualized using DCF-DA which produced green fluorescence. The DCF-DA fluorescence was negligible in the root tips of control plants, whereas it increased markedly under Al stress. The level of H_2_O_2_ was higher in both the resistant and sensitive genotypes; however, when both were compared, low fluorescent signal was observed in roots of resistant breeding lines (L-7903 and L-4602), whereas intense green fluorescence was found at roots tips in sensitive cultivars (BM-4 and L-4147), although they were treated with same level of Al stress *i*.*e*. 74 and 148 μM Al concentration (S5 Fig). Similar to cultigens, wild resistant accessions exhibited less green fluorescence when compared to sensitive wilds, indicating less H_2_O_2_ production under both the treatment conditions.

#### Effect of Al stress on lipid peroxidation

Lipid peroxidation was estimated biochemically as concentration of TBARS. There was a great variation for TBARS concentration among the four cultigens and two wilds under Al stressed conditions, indicating intraspecific genotypic variability. In general, treatment with higher Al concentration resulted in significantly high TBARS in both resistant and sensitive genotypes; however, sensitive cultigens showed more TBARS concentration as compared to resistant lines (S4 Fig). The concentration of TBARS in resistant wild accession, ILWL-185 in both roots and shoots at 148 μM was lowest when compared to all the genotypes (cultigens as well as wilds). In roots, at 148 μM Al concentration, TBARS concentration was highest in sensitive wild, ILWL-436 and in shoots the concentration was comparable to sensitive cultigens.

#### Effects of Al stress on antioxidant systems

**Fig 7:**
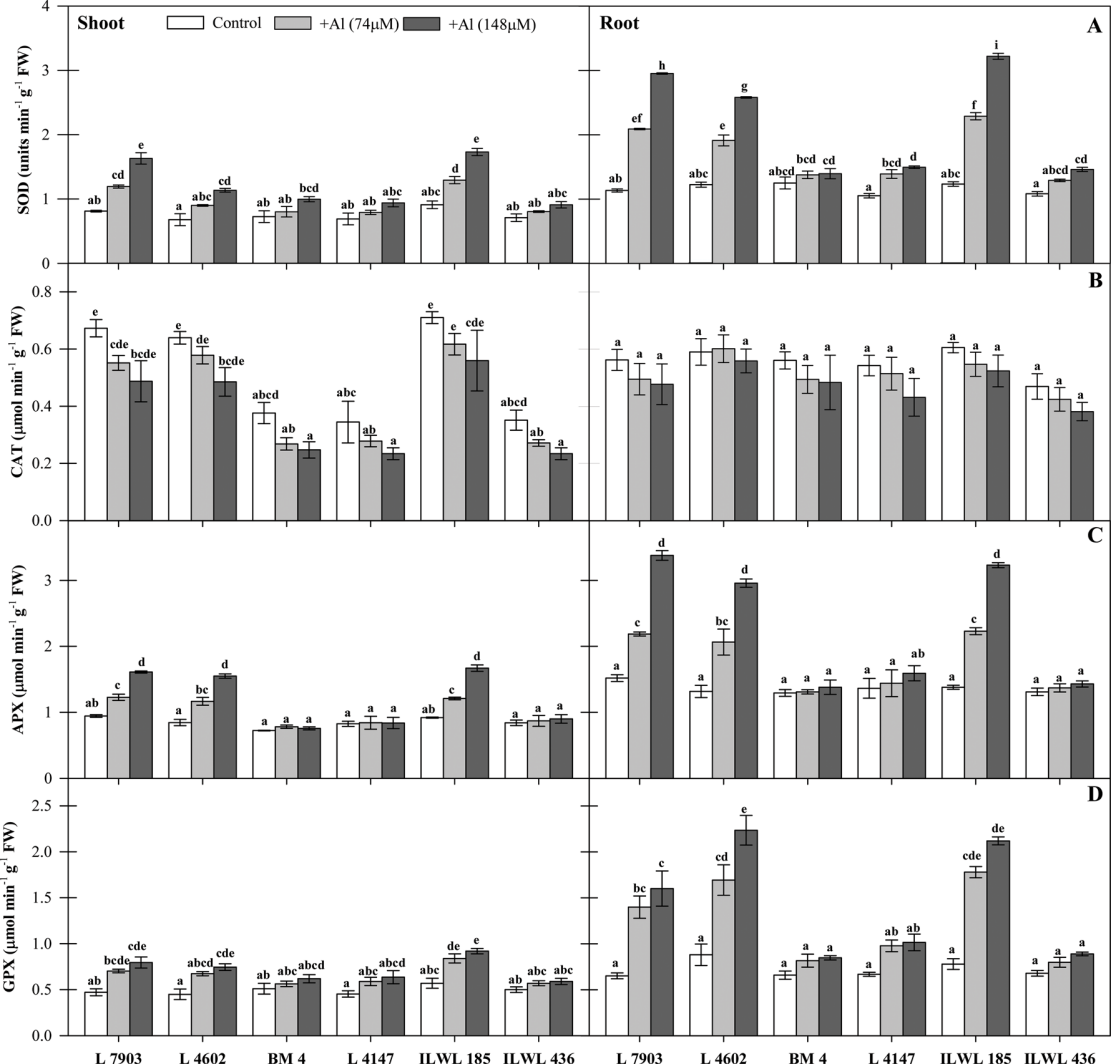
Antioxidant enzyme activity of A- SOD, B- CAT, C- APX and D-GPX in four lentil genotypes: Resistant breeding lines (L-7903, L-4602), Sensitive cultivars (BM-4, L-4147) and two wild accessions: Resistant (ILWL-185), Sensitive (ILWL-436) under Al stress (74 and 148μM Al) conditions after 48 h exposure. Means with the same small letters for each part of the plant do not statistically differ by the Tukey test at P≤0.05.

SOD, APX, GPX and CAT activities were estimated in both roots and shoots. The results revealed that Al stress affected activities of all the antioxidant enzymes variably. Higher SOD activity was observed in roots and shoots of both resistant and sensitive genotypes under Al stress; however, it was more prominent in roots than in shoots. Increment in SOD activity in cultigens was more in resistant breeding lines (L-7903 and L-4602), while sensitive cultivars (BM-4 and L-4147) showed less increase in SOD activity under Al stress as compared to the controls. The increase in enzyme activity was more in roots of L-7903 and L-4602 (61.5 and 52.5%) than in BM-4 and L-4147 (10.4 and 29.7%) genotypes at 148 μM concentration of Al (Fig 7a). The most prominent SOD activity was observed in roots of resistant wild accession, ILWL-185 (61.6 at 148 μM concentration of Al) while the sensitive wild accession, ILWL-436 (25.9 at 148 μM concentration of Al) showed lowest activity among all the genotypes. On the other hand, catalase activity decreased significantly under Al stress in both the resistant and sensitive genotypes when compared to their respective controls. With increasing levels of Al stress, a concomitant decline in catalase activity was observed in roots and shoots of both the resistant and sensitive genotypes. Seedlings grown under 148 μM Al concentration showed about 5.6 and 17.9% decline in catalase activity in roots and 38.0 and 31.8% decline in activity in shoots of breeding lines, L-7903 and L-4602, respectively (Fig 7b). In case of wild accessions, resistant one (ILWL-185) showed highest CAT activity, with reduction of 26.9% in shoots when compared to resistant cultigens, while the activity in roots (with reduction over the control of 15.7%) was equivalent to resistant cultigens. Similar to SOD, APX activity increased in roots and shoots of resistant breeding lines, L-7903 and L-4602 as well as resistant wild, ILWL-185. The increase in activity of APX was higher in roots as compared to shoots and the increase was more in resistant breeding lines, L-7903 and L-4602 (50.0 and 55.5%) than in sensitive cultivars, BM-4 and L-4147 (6.3 and 14.3%) at 148 μM Al concentration as compared to their controls. Similar to roots, APX activity increased in shoots of all the four cultigens, but, L-7903 and L-4602 showed higher increase (41.4 and 45.4%) as compared to marginal increase of 4.5 and 1.4% in BM-4 and L-4147, respectively at 148 μM Al concentration. Resistant wild, ILWL-185 also showed significant increase in APX activity in both roots (57.3% at 148 μM Al concentration) and shoots (44.9% at 148 μM Al concentration), while the sensitive wild, ILWL-436 showed very less increase in activity (8.4% in roots and 6.7% in shoots at 148 μM Al concentration) which was just comparable to the control (Fig 7c). Al stress also caused significant (P< 0.05) increase in GPX activity in all the genotypes. The genotypes L-7903 and L-4602 showed higher increase in GPX activity (59.3 and 60.6%) as compared to BM-4 and L-4147 (22.2 and 34.1%) at 148 μM Al concentration. As such, in the shoots, GPX activity increased by 40.7 and 39.7% in L-7903 and L-4602 (resistant) and 17.6 and 28.8% in BM-4 and L-4147 (sensitive) genotypes, respectively at 148 μM Al as compared to their controls. The wild accessions also showed significant increase in GPX activity in roots which was very high in resistant genotype (63.2% at 148 μM Al concentration). Alternatively, the sensitive one showed very less increase in GPX activity (23.6% at 148 μM Al concentration) (Fig 7d).

#### Al-induced secretion of organic acids

**Fig 8:**
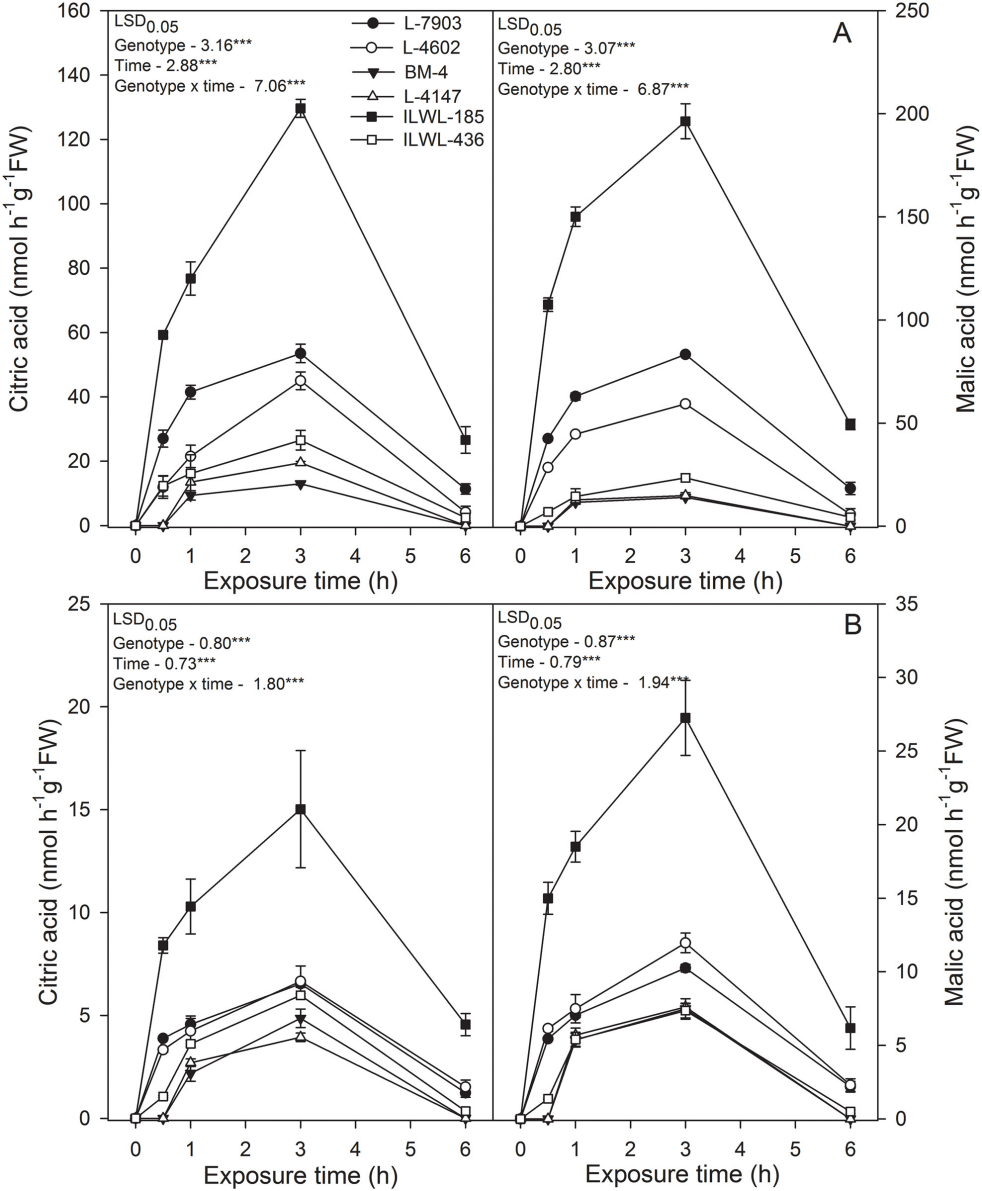
Organic acids exudation in roots of lentil genotypes: Resistant breeding lines (L-7903, L-4602), Sensitive cultivars (BM-4, L-4147) and wild accessions: Resistant (ILWL-185), Sensitive (ILWL-436) after exposure to 74 μM (A) and 148μM (B) Al concentration. LSD 0.05 = least significant difference value at P <0.05 for genotype, time and genotype x time interaction (n = 3). *** indicates significance at P<0.001.

There were two peaks corresponding to citrate (retention time 8.6 min) and malate (retention time 10.2 min) in HPLC profile after treatment with 74 and 148 μM Al (pH 4.5) for 30 min, 1 h, 2 h, 3 h and 6 h (Fig 8) and organic acids were not detected in the absence of Al. Exposure to Al caused increased secretion of citrate and malate from roots in all the genotypes, but the amount of secretion was less in sensitive genotypes (BM-4, L-4147, ILWL-185) than resistant ones (L-7903, L-4602 and ILWL-436). Also, in resistant genotypes, malate was detected at high level in comparison to citrate in the nutrient solution under Al treatment. The secretion of both the organic acids was highest in wild resistant accession, ILWL-185 at both treatment conditions (74 and 148 μM Al). The secretion of citrate and malate was detected in 1–3 h after exposure to 74 to 148 μM AlCl_3_.6H_2_O (pH 4.5) (Fig 8) and after 3 h exposure to Al, no significant changes were detected on the secretion of citrate and malate. These results suggested that the secretion of high levels of citrate and malate required approximately 3 h of Al stimulation.

### Field evaluation of selected genotypes in acidic conditions

**Fig 9:**
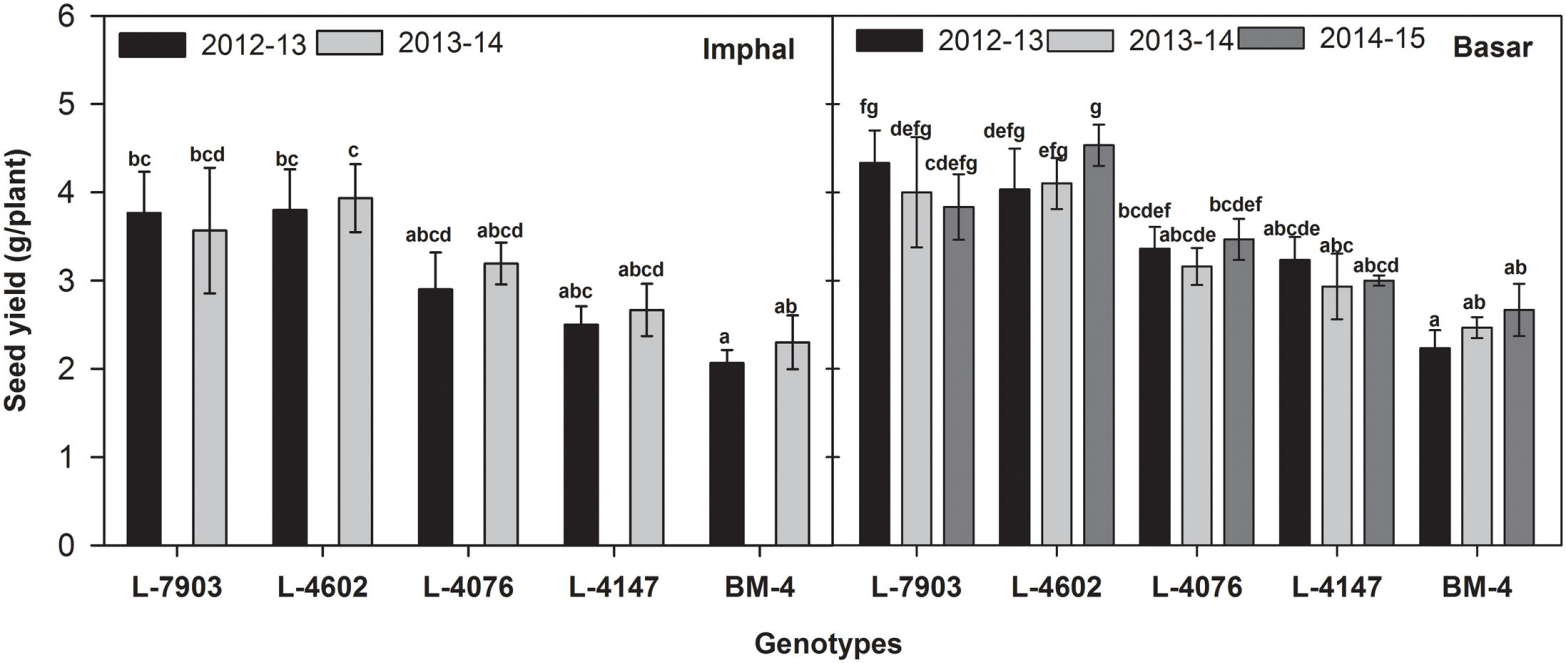
Seed yield of lentil genotypes grown under low pH condition at Imphal, Manipur, India (pH 4.8) and Basar, Arunachal Pradesh, India (pH 5.1) during 2012–13, 2013–14 and 2014–15. Data shown are mean ± SEm. Bars that do not share common letters are significantly different by Duncan’s post hoc test at P<0.05.

The field experiments under acidic conditions were conducted during 2012–13 and 2013–14 at Imphal, Manipur, India and during 2012–13, 2013–14 and 2014–15 at Basar, Arunanchal Pradesh, India for evaluation of Al resistance in selected genotypes. The resistant breeding lines (L-7903 and L-4602) showed 3.77 g and 3.57 g; 3.80 g and 3.93 g seed yield per plant during 2012–13 and 2013–14 at Imphal, Manipur, India, respectively. These two breeding lines showed 4.33 g, 4.00 g and 3.83 g; 4.03 g, 4.10 g and 4.53 g seed yield per plant during 2012–13, 2013–14 and 2014–15, respectively at Basar, Arunanchal Pradesh, India. On the other hand, sensitive genotypes (BM-4 and L-4147) recorded the seed yield per plant of 2.07 g and 2.34 g; 2.50 g and 2.67 g during 2012–13 and 2013–14, respectively at Imphal, Manipur, India and 2.23 g, 2.47 g and 2.67 g; 3.23 g, 2.93 g and 3.00 g during 2012–13, 2013–14 and 2014–15, respectively at Basar, Arunanchal Pradesh, India (Fig 9). At the two locations *i*.*e*. Imphal and Basar, significant correlation was found between seed yield and RRG (r = 0.96 and r = 0.94, respectively).

## Discussion

Aluminium is easily absorbed by plants mainly through the root system and thereafter displays its toxicity symptoms. Root growth inhibition is one of the indicators of Al sensitivity which may occur due to disruption of root cell physiological processes [5]. In the present study also, the inhibition of the roots was much more in sensitive genotypes than in the resistant ones. Such growth inhibition under Al stress may be the result of more Al accumulation in the roots. This result was supported by RRG after haematoxylin staining without Al stress. This trait has been suggested as an indicator of Al resistance in different crops like wheat, coffee, sorghum etc. [49, 50, 51]. On the basis of this important trait, all the 285 genotypes were grouped into Al resistant, moderately resistant and sensitive categories. From all these genotypes, couple of most contrasting cultigens and a pair of contrasting wilds were further selected to study Al resistance mechanism in lentil. Also, for improving Al tolerance in cultigens, it is essential to check the genetic dissimilarity among the contrasting genotypes which could act as potential parents in future Al resistance breeding programmes and the crossable resistant wilds could provide important Al resistance genes for introgression into cultigens. Therefore, in this study an attempt was made to categorize all the 285 genotypes including breeding lines, cultivars, landraces, germplasm collections as well as wilds, based on their molecular diversity and then to correlate them with their Al resistance reactions under different experimental conditions. Based on cluster analysis, out of 7 resistant breeding line, germplasm collection, landraces, *viz*. L-7903, L-4602, LC-284-116, PDL-1, ILL-10794, IG-129319 and PSL-7; 3 were grouped in cluster C11, two were grouped in C9 and one fall into C1 whereas among 13 sensitive, breeding lines, germplasm collection, landraces, cultivars *viz*. BM-4, L-4147, IG-109039, IG-112128, IG-129302, IG-130219, L-4590, IG-9, IG-936, ILL-10960, LC-300-6, PSL-1 and IG-5320, 7 were grouped into cluster C11, two were in C6 while other four were scattered in clusters C5, C8, C9 and C10. All the 4 wild resistant accessions *viz*. ILWL-185, ILWL-340, ILWL-3 and ILWL-29 were grouped together in cluster C4. Also, out of 7 sensitive wild accessions *viz*. IG-136620, ILWL-125, ILWL-227, ILWL-398(A), ILWL-415, ILWL-44, ILWL-436, first six were grouped together in cluster C3 and last one is separated into C6 (Fig 1). This suggested that grouping of wild accessions was on the basis of Al resistance reactions, while cultigens showed lesser Al tolerance grouping pattern. Similarly, separation of wild accessions from cultigens in lentil has been reported in some recent studies [52, 12]. Further, among 4 resistant wilds, ILWL-3 belongs to spp. *L*. *orientalis* which is crossable with cultigens and therefore can be utilized for Al tolerance improvement through breeding programs.

Growth parameters like reduction in root length and shoot length was low in the genotypes of cluster 4 and 1 which was comparatively high in those of other clusters. These clusters consisted of resistant genotypes mainly. The genetic diversity within wild separated itself from the cultivars and ‘ILL’ series of cultigens which were mainly germplasm collections from ICARDA were grouped together in cluster C10 (Fig 1). Similarly, distinction of wild types from cultivars has previously been reported in many other crops including pigeonpea, pearl millet etc. [53, 54]. These results were also supported by both the population structure developed using STRUCTURE software and Jaccard’s similarity index obtained from NTSYS-pc software. Further, most of the wild accessions were clustered according to their subspecies as *L*. *culinaris* ssp. *ervoides* were mostly clustered into C3, similarly cluster 6 mostly comprised of *L*. *culinaris* ssp. *odemensis* and C7 has mostly genotypes of *L*. *culinaris* ssp. *orientalis*. Characterization of wild lentil accessions on the basis of their subspecies was also reported by Dikshit *et al*. [52]. On the basis on population structure, genotypes were distinguished into 3 population groups, where wilds were separated from cultigens. Also, 125 genotypes were assigned to admixtures reflecting hybridization and migration events between the populations and possible gene flow between distinct populations. These admixes can be further used for Admixture mapping following association study. Pfaff *et al*. have reported that Admixture mapping is a potentially powerful method for the identification of genes that elude other mapping methods [55]. Jaccard’s similarity index which correlated genetic similarity and RRG after staining had well separated the six selected resistant and sensitive genotypes (cultigens as well as wilds) (Fig 2). This suggested that such trait among different genotypes can be predicted based on genetic similarity by SSR markers. Similarly, separation of drought resistant and sensitive genotypes through Jaccard’s similarity index has been reported by Singh *et al*. [12].

The six selected genotypes *viz*. L-7903, L-4602 (resistant breeding lines); BM-4, L-4147 (sensitive cultivars); ILWL-185 (resistant wild), ILWL-436 (sensitive wild) were then used to study different component mechanisms for Al resistance in lentil. The findings showed less Al content in roots and shoots in resistant lines as compared to sensitive ones (Fig 4). Less retention of Al in roots, restricting its transport to shoot in resistant lines (L-7903 and L-4602) suggests that efficient exclusion mechanism might be involved in these genotypes. The resistant genotypes either restricted Al absorption through roots or detoxify it after absorption [56]. Staining with morin and haematoxylin fluorescent dyes, have been used in the past to detect the presence of Al as they are relatively simple and quick tools to examine Al^3+^ localization and accumulation in root tissues [57, 37, 25]. When a plant is grown under high Al concentrations, Al first enters in root tissues of cell wall (upper layers) and it is well evidenced that primary Al accumulation in root tissues is localized in cell wall [58]. In this study, morin staining demonstrated lower accumulation of Al in cell dividing and elongation zones of roots in Al resistant lines compared to sensitive ones in both cultigens and wilds (Fig 5). These results suggest that Al-resistant roots have developed a mechanism to restrict accumulation of Al in this region. Al entering the root during the 48 h exposure period had penetrated the endodermis and cortex of BM-4, L-4147 and ILWL-436, but not in L-7903, L-4602 and ILWL-185. This clearly indicates the existence of barrier mechanism in resistant lines that restricts Al^3+^ penetration into the endodermis and cortex up to certain Al stress levels. Similar staining pattern was observed in root tips of wheat by Andrade *et al*. [59]. Ahn *et al*. reported that most of the Al detected by morin was located in cell walls of squash root cells during the first 3 h of treatment [60]. Vázquez *et al*. observed the presence of Al in cell walls and vacuole of maize root tip cells after 4 h of Al treatment [61].

Callose accumulation in roots has also been known as a sensitive indicator of A1 toxicity and can be readily detected by fluorescence microscopy [62, 36]. In controls (absence of Al), resistant and sensitive genotypes showed a negligible fluorescence, whereas strong fluorescence signals (callose accumulation) were detected in roots after exposure to 74 and 148 μm Al for 48 h. Callose was observed slightly lower in resistant breeding lines (L-7903, L-4602 and ILWL-185) than in sensitive cultivars (BM-4, L-4147 and ILWL-436) (Fig 6). This suggests that callose accumulation in Al-treated roots of resistant lines was less in comparison to accumulation in sensitive ones. Production of higher ROS and fatty acid oxidation of root plasma membrane is well documented under Al toxicity [63, 64]. In this study, level of ROS induced H_2_O_2_ production was estimated through florescence imaging and lower level of H_2_O_2_ was found in resistant genotypes in comparison to sensitive ones (S5 Fig). Lipid peroxidation has also been suggested as an indicator of Al sensitivity in plants [65]. Lipid peroxidation in the roots of all the six genotypes under Al stress were found to be more pronounced in the root tips of sensitive genotypes than resistant ones. Basu *et al*. has also reported similar increase in the level of malondialdehyde (MDA) /TBARS and directly correlated it with the inhibition of root growth in *Brassica napus* [66].

Plants possess several enzymatic and non-enzymatic antioxidant mechanisms to overcome the oxidative stress caused by over production of ROS [67]. In this study, the role of antioxidant enzymes (SOD, APX, GPX and CAT) under Al stress in detoxification of ROS was well observed. There was induction in SOD, APX and GPX activity in all the genotypes under Al stress in both roots and shoots. The activity of all these enzymes was higher in roots as compared to shoots, which was associated with higher Al concentration in the roots [68]. Resistant genotypes showed significant increase in SOD activity at both the levels of Al stress *i*.*e*. 74 and 148 μM, on the other hand, sensitive genotypes showed no significant changes in SOD activity. The highest increase in SOD activity over the control was observed in roots of wild resistant accession, ILWL-185 (Fig 7a). Boscolo *et al*. have reported similar Al-induced activity of SOD in different crops [69]. APX and GPX activity also showed similar patterns in all the genotypes under Al stress (Fig 7c and 7d). The activity of APX was shown to be enhanced by Al in crops like *Cucurbita pepo* and rice [70, 68]. Furthermore, Singh *et al*. have used GPX activity as biomarker to evaluate the intensity of Al stress in chickpea [71]. In contrast to the activities of these three enzymes, CAT activity decreased in shoots of all the six genotypes under Al stress where sensitive genotypes showed higher reduction compared to resistant ones. On the other hand, CAT activity in roots has shown no significant changes under Al stress in sensitive as well as tolerant lines (Fig 7b). In contrast to this, there are various reports where CAT activity was shown to be enhanced under Al stress in plants like pea and soybean [72, 73]. In present study, reduction in the CAT activity in shoots under Al stress suggests that CAT may be involved in H_2_O_2_ degradation [69] and the decreased CAT activity can serve as an intrinsic defence mechanism to resist Al-induced oxidative damage in lentil.

Plants uses different types of strategies to mitigate adverse effects of Al toxicity where exclusion is most widely accepted one [74, 75]. Exclusion may involve either exudation of chelating ligands or formation of pH barrier at root apoplasm or Al efflux among which, exclusion through chelating ligands is reported as most dominant mechanism in many plant species [76, 77]. Organic acids like citrate, malate and oxalate have been reported to be exudated at rhizosphere and apoplast under Al stress [78]. These organic acids can form stable, nontoxic complexes with Al in the rhizosphere, which prevent binding of Al to extra- cellular and intra-cellular substances of the roots [79, 80]. In this study, secretion of citrate and malate from roots in response to Al stress was observed, where the amount of malate secretion was found to be higher in comparison to citrate at 74 and148 μM Al concentration (Fig 8). Secretion of both citrate and malate was higher in Al resistant cultigens as well as wild than in sensitive ones and it was observed that higher secretion of citrate and malate in resistant genotypes was during first 3h of Al exposure, thereafter the secretion of both the organic acids declined. The secretion of both the organic acids was highest in the wild resistant accession, ILWL-185. This pattern of secretion of exudates has been suggested as pattern II by Ma *et al*. [80]. Similar pattern of Al-induced organic acid secretion has been reported in tora, soybean, common bean and rice bean [81, 82, 83, 84]. In this study, the reduction in amount of citrate and malate secretion after 3h of Al stress treatment suggested that Al exposure for longer duration may probably influence the mechanism involved in secretion.

In this study, field experiments for Al resistance conducted at highly Al toxicity affected areas in India. Lentil genotypes at Imphal, Manipur, India location had low seed yield per plant than those grown at Basar, Arunachal Pradesh, India. The higher seed yield at Basar, Arunachal Pradesh, India may be because of slightly higher pH (5.1) in this region. At both the locations, L-7903 and L-4602 (resistant breeding lines), had higher seed yield per plant than BM-4 and L-4147 (sensitive cultivars) under low pH conditions (Fig 9). It was also noted that RRG is highly correlated with seed yield at both the locations. Thus, while screening of a large number of genotypes for Al resistance, it is recommended that selection of genotypes be based on RRG. Selected resistant breeding lines (L-7903, L-4602) and wild (ILWL-185) accessions can be used in breeding programme for development of aluminium resistant genotypes. Under all the experimental conditions implied in this study, wild resistant accessions showed higher or similar results to that of resistant cultigens. Also, wild accessions were grouped into one population group as indicated by both population structure and cluster analysis. This could be due to maintenance of originality of their genetic constituents whereas in cultigens there is extensive inbreeding and domestication over the decades which have distorted their genetic constituents.

## Conclusions

The important acquisition in this study could be applied to lentil breeding programs for improving Al resistance. Improvement in aluminium tolerance can be achieved by crossing contrasting parental genotypes from different SSR clusters. Further crossing between some of the resistant and sensitive cultigens which are grouped together in cluster C11 should be avoided in breeding programmes for Al tolerance because of their low genetic dissimilarity. Also, physiological and biochemical mechanisms/traits for Al stress tolerance described in this study in lentil will allow the design of better techniques for reducing the period of developing Al stress resistant genotypes in future breeding programs. Various component traits/ mechanism evaluated in contrasting lentil genotypes like root growth, Al accumulation/ compartmentation and exudation of organic acids, can be considered felicitous for selection of Al stress resistant lentil genotypes and can be exploited in future breeding programs for mapping and introgression of Al resistance gene/ quantitative trait loci (QTL) for development of Al resistant cultivars for acidic soils.

## Supporting Information

S1 Fig Electrophoretic profile of PCR amplified products of SSR marker PLC_100 in 48 cultivars. Base pairs (bp), 100bp DNA Ladder (L).
(TIF)

S2 Fig Electrophoretic profile of PCR amplified products of SSR marker PBA_LC_1551 in 48 wild genotypes. Base pairs (bp), 100bp DNA Ladder (L).
(TIF)

S3 Fig Evanno plot describing estimation of cultigens and wild genotypes of genus *Lens* using LnP (D) derived Δ k for k from 1 to 15. (TIF)

S4 Fig Changes in TBARS contents for resistant breeding lines (L-7903 and L-4602), sensitive cultivars (BM-4 and L-4147), resistant wild accession (ILWL-185) and sensitive wild accession (ILWL-436) under Al stress (74 and 148μM Al) conditions after 48 h exposure. Means with the same small letters for each part of the plant do not statistically differ by the Tukey test at P≤0.05.
(TIF)

S5 Fig Histochemical detection of H_2_O_2_ through–fluorescent microscope image in root tips of lentil seedling of control, 74μM and 148 μM Al. Bar in each figure represent 1 mm.
(TIF)

## References

[1] FAO (Food and Agricultural Organization). 2014; Available: http://www.fao.org.

[2] Singh D, Dikshit HK, Singh R. Variation of Aluminium tolerance in lentil. Plant Breed. 2012; 131: 751–761.

[3] Mandal AB, Basu AK, Roy B, Sheeja TE, Roy T. Genetic management for increased tolerance to aluminium and iron toxicities in rice- A review. Indian J Biotechnol. 2004; 3: 359–368.

[4] Singh D, Singh NP, Chauhan SK, Singh P. Developing aluminium tolerant crop plants using biotechnological tools. Curr Sci. 2011; 100: 1807–1813.

[5] Kochian LV, Pineros MA, Hoekenga OA. The physiology, genetics and molecular biology of plant aluminium resistance and toxicity. Plant Soil. 2005; 274: 175–195.

[6] Wissemeier AH, Klotz F, Horst WJ. Aluminium induced callose synthesis in roots of soybean (*Glycine max* L). J Plant Physiol. 1987; 129: 487–492.

[7] Zhang G, Hoddinott J, Taylor GJ. Characterization of 1,3-β-D-glucan (callose) synthesis in roots of *Triticum aestivum* in response to Aluminium toxicity. J Plant Physiol. 1994; 144: 229–234.

[8] Butare L, Rao I, Lepoivre P, Cajiao C, Polania J, Cuasquer J, et al Phenotypic evaluation of interspecific recombinant inbred lines (RILs) of *Phaseolus* species for aluminium resistance and shoot and root growth response to aluminium–toxic acid soil. Euphytica. 2011.

[9] Govindaraj M, Vetriventhan M, Srinivasan M. Importance of genetic diversity assessment in crop plants and its recent advances: an overview of its analytical perspectives. Genet Res Int. 2015 10.1155/2015/431487 PMC4383386 25874132

[10] Blair MW, López-Marín HD, Rao IM. Identification of Aluminium resistant Andean common bean (*Phaseolus vulgaris* L.) genotypes. Braz J Plant Physiol. 2009; 21(4): 291–300.

[11] Sharma M, Trofimova M, Sharma V, Tripathi BN. Genotypic variation to aluminium sensitivity in chickpea depends on its ability to efficiently accumulate nitrate. Adv Agron Plant Sci. 2015 1(1): 1–12.

[12] Singh D, Singh CK, Tomar RSS, Taunk J, Singh R, Maurya S, et al Molecular assortment of *Lens* species with different adaptations to drought conditions using SSR markers. PLoS ONE. 2016 10.1371/journal.pone.0147213 PMC4726755 26808306

[13] Singh D, Singh CK, Tomar RS, Chaturvedi AK, Shah D, Kumar A, et al Exploring genetic diversity for heat tolerance among lentil (*Lens culinaris* Medik.) genotypes of variant habitats by simple sequence repeat markers. Plant Breed. 2016 10.1111/pbr.12341

[14] Sivaguru M, Horst WJ, Eticha D, Matsumoto H. Aluminium inhibits apoplastic flow of high-molecular weight solutes in root apices of *Zea mays* L. J Plant Nut Soil Sci. 2006; 169: 679–690.

[15] Gille G, Sigler K. Oxidative stress and living cells. Folia Microbiologica. 1995; 40: 131–152. 8851559 10.1007/BF02815413

[16] Romero–Puertas MC, Corpas FJ, Rodriguez–Serrano M, Gomez M, del Río LA, Sandalio LM. Differential expression and regulation of antioxidative enzymes by Cd in pea plants. J Plant Physiol. 2007; 164: 1346–1357. 17074418 10.1016/j.jplph.2006.06.018

[17] Gratao PL, Polle A, Lea PJ, Azevedo RA. Making the life of heavy metal–stressed plants a little easier. Funct Plant Biol. 2005; 32: 481–494. 10.1071/FP05016 32689149

[18] Apel K, Hirt H. Reactive oxygen species: Metabolism, oxidative stress, and signal transduction. Ann Rev Plant Biol. 2004; 55: 373–99. 15377225 10.1146/annurev.arplant.55.031903.141701

[19] Tamás L, Simonovicová M, Huttová J, Mistrík I. Aluminium stimulated hydrogen peroxide production of germinating barley seeds. Environ Exp Bot. 2004; 51: 281–288.

[20] Brunner I, Sperisen C. Aluminium exclusion and aluminium tolerance in woody plants. Frontiers Plant Sci. 2013; 4: 172–184. 10.3389/fpls.2013.00172 PMC3679494 23781222

[21] Ma JF, Taketa S, Yang ZM. Aluminium tolerance genes on the short arm of chromosome 3R are linked to organic acid release in triticale. Plant Physiol. 2000; 122: 687–694. 10712531 10.1104/pp.122.3.687 PMC58903

[22] Pineros MA, Magalhaes JV, Alves VMC, Kochian LV. The physiology and biophysics of an Aluminium tolerance mechanism based on root citrate exudation in maize. Plant Physiol. 2002; 129: 1194–1206. 12114573 10.1104/pp.002295 PMC166513

[23] Singh D, Singh NP. Genetic and molecular mechanisms of aluminium tolerance in crop plants. Biotech Today. 2012; 2(2): 52–55.

[24] Simon L, Smalley TJ, Jones JB, Lasseigne FT. Aluminium toxicity in tomato: Part I Growth and mineral nutrition. J Plant Nut. 1994; 17: 293–306.

[25] Polle E, Konzak CF, Kittrick JA. Visual detection of aluminium tolerance levels in wheat by hematoxylin staining of seedlings roots. Crop Sci. 1978; 18: 823–827.

[26] Doyle JJ, Doyle JL. Isolation of plant DNA from fresh tissue. Focus. 1990; 12: 13–15.

[27] Hamwieh A, Udupa SM, Choumane W, Sarker A, Dreyer F, Jung C, et al A genetic linkage map of *Lens* sp. based on microsatellite and AFLP markers and the localization of *Fusarium* vascular wilt resistance. Theor Appl Genet. 2005; 110: 669–677. 15650814 10.1007/s00122-004-1892-5

[28] Kaur S, Cogan N, Amber S, Noy D, Butsch M, Froster JW, et al EST-SNP discovery and dense genetic mapping in lentil (Lens culinaris Medik.) enable candidate gene selection for boron tolerance. Theor Appl Genet. 2014; 127(3): 703–713. 10.1007/s00122-013-2252-0 24370962

[29] Jain N, Dikshit HK, Singh D, Singh A, Kumar H. Discovery of EST-derived microsatellite primers in the legume *Lens culinaris* (Fabaceae). Appl Plant Sci. 2013 10.3732/apps.1200539 PMC4103130 25202567

[30] Liu K, Muse S. Power Marker: new genetic data analysis software, version 2.7. 2004; Available: http://www.powermarker.net.

[31] Tamura K, Dudley J, Nei M, Kumar S. Molecular evolutionary genetics analysis (MEGA) software version 40. Mol Biol Evol. 2007; 24: 1596–1599. 17488738 10.1093/molbev/msm092

[32] Pritchard JK, Matthew S, Peter D. Inference of population structure using multilocus genotype data. Genetics. 2000; 155: 945–959. 10835412 10.1093/genetics/155.2.945 PMC1461096

[33] Earl DA, Von H, Bridgett M. STRUCTURE HARVESTER: a website and program for visualizing STRUCTURE output and implementing the Evanno method. Conserv Genet Resour. 2012; 4: 359–361.

[34] Rohlf FJ. NTSYS-pc: numerical taxonomy and multivariate analysis system, version 2.2 Exeter Software, Setauket, NY 2000; Available: http://www.exetersoftware.com/cat/ntsyspc/ntsyspc.html.

[35] Kauss H. Callose and callose synthase In: Gurr S.J., McPherson M.J., Bowles D.J., editors. Molecular Plant Morphology: A Practical Approach. Oxford University Press, New York; 1992 pp. 1–8.

[36] Singh D, Dikshit HK, Kumar A. Aluminium tolerance in lentil (*Lens culinaris* Medik) with monogenic inheritance pattern. Plant Breed. 2015; 134: 105–110.

[37] Tice KR, Parker DR, Demason DA. Operationally defined apoplastic and symplastic Aluminium fractions in root tips of Aluminium–intoxicated wheat. Plant Physiol. 1992; 100: 309–318. 16652962 10.1104/pp.100.1.309 PMC1075553

[38] Heath RL, Packer L. Photoperoxidation in isolated chloroplasts. I. Kinetics and stoichiometry of fatty acid peroxidation. Arch Biochem Biophy. 1968; 125: 189–198. 10.1016/0003-9861(68)90654-1 5655425

[39] Aebi H. Catalase *in vitro*. Methods Enzymol. 1984; 105: 121–126. 6727660 10.1016/s0076-6879(84)05016-3

[40] Dhindsa RS, Plump–Dhindsa P, Thorpe TA. Leaf senescence: Correlated with increased levels of membrane permeability and lipid peroxidation, and decreased levels of superoxide dismutase and catalase. J Exp Bot. 1981; 32: 93–101.

[41] Nakano Y, Asada K. Hydrogen peroxide is scavenged by ascorbate–specific peroxidase in spinach chloroplasts. Plant Cell Physiol. 1981; 22: 867–880.

[42] Castillo FJ, Penel C, Greppin H. Peroxidase release induced by ozone in Sedum album leaves, Plant Physiol. 1984; 74: 846–851. 16663520 10.1104/pp.74.4.846 PMC1066779

[43] Rao MV, Paliyath G, Ormrod DP. Ultraviolet–B and ozone–induced biochemical changes in antioxidant enzymes of *Arabidopsis thaliana*. Plant Physiol. 1996; 110: 125–136. 8587977 10.1104/pp.110.1.125 PMC157701

[44] Zhao Z, Ma JF, Sato K, Takeda K. Differential Al resistance and citrate secretion in barley (*Hordeum vulgare* L.). Planta. 2003; 217: 794–800. 12734756 10.1007/s00425-003-1043-2

[45] Walkley A, Black IA. An examination of degtjareff method for determining soil organic matter and a proposed modification of the chromic acid titration method. Soil Sci. 1934; 37: 29–37.

[46] Jackson ML. Soil Chemical Analysis, Prentice Hall of India, New Delhi, India; 1973.

[47] Bray RH, Kurtz LT. Determination of total, organic, and available forms of phosphorus in soil. Soil Sci. 1945; 59: 39–45.

[48] Mehlich A. New extractant for soil test evaluation of phosphorus, potassium, magnesium, calcium, sodium, manganese and zinc. Commun Soil Sci Plant Anal. 1978; 9: 477–492.

[49] Darkó É, Bakos F, Szakács É, Dulai S, Barnabás B. Long-term phytotoxic effects of aluminium on Al susceptible and Al-tolerant genotypes of wheat selected from microspores. Acta Biologica Szegediensis. 2002; 46: 87–88.

[50] Braccini MCL, Martinez HEP, Silva EAM, Braccini AL, Scapim CA. Plant growth and root hematoxylin staining to evaluate Aluminium toxicity tolerance of coffee genotypes. Rev Bras Ciênc Solo. 2000; 24(1): 59–68.

[51] Anas, Yoshida T. Screening of Al-tolerant sorghum by hematoxylin staining and growth response. Plant Prod Sci. 2000; 3(3): 246–253.

[52] Dikshit HK, Singh A, Singh D, Aski MS, Prakash P, Jain N, et al Genetic diversity in *Lens* species revealed by EST and genomic simple sequence repeat analysis. Plos One. 2015 10.1371/journal.pone.0138101 PMC4575128 26381889

[53] Yang S, Pang W, Ash G, Harper J, Carling J, Wenzl P, et al Low level of genetic diversity in cultivated pigeonpea compared to its wild relatives is revealed by diversity arrays technology. Theor Appl Genet. 2006; 113: 585–595. 16845522 10.1007/s00122-006-0317-z

[54] Mariac C, Luong V, Kapran I, Mamadou A, Sagnard F, Deu M, et al Diversity of wild and cultivated pearl millet accessions (*Pennisetum glaucum* L.) in Niger assessed by microsatellite markers. Theor Appl Genet. 2006; 114: 49–58. 17047913 10.1007/s00122-006-0409-9

[55] Pfaff CL, Parra EJ, Bonilla C, Hiester K, McKeigue PM, Kamboh MI, et al Population structure in admixed populations: effect of admixture dynamics on the pattern of linkage disequilibrium. Am J Hum Genet. 2001; 68: 198–207. 11112661 10.1086/316935 PMC1234913

[56] Fageria NK, Baligar VC, Wright RJ. Aluminium toxicity in crop plants. J Plant Nutr. 1988; 11: 303–319.

[57] Eggert DA. Use of morin for fluorescent localization of aluminium in plant tissues. Stain Technol. 1970; 45: 301–303. 5490089 10.3109/10520297009067806

[58] Hossain M, Zhou MX, Mendham NJ. A reliable screening system for aluminium tolerance in barley cultivars. Australian J Agri Res. 2005; 56: 475–482.

[59] Andrade LRM, Ikeda M, Ishizuka J. Stimulation of organic acid excretion by roots of Aluminium–tolerant and aluminium sensitive wheat varieties under aluminium stress. Braz J Plant Physiol. 1997; 9: 27–34.

[60] Ahn SJ, Sivaguru M, Chung GC, Rengel Z, Matsumoto H. Aluminium–induced growth inhibition is associated with impaired efflux and influx of H^+^ across the plasma membrane in root apices of squash (*Cucurbita pepo*). J Environ Quality. 2002; 53: 1959–1966. 10.1093/jxb/erf049 12177136

[61] Vazquez MD, Poschenrieder C, Corrales I, Barcelo J. Change in apoplastic aluminium during the initial growth response to aluminium by roots of a tolerant maize variety. Plant Physiol. 1999; 119: 435–444. 9952438 10.1104/pp.119.2.435 PMC32119

[62] Alvim MN, Ramos FT, Oliveira DC, Isaias RMS, França MGC. Aluminium localization and toxicity symptoms related to root growth inhibition in rice (*Oryza sativa* L.) seedlings. J Bioscience. 2012; 37: 1079–1088. 10.1007/s12038-012-9275-6 23151797

[63] Sun P, Tian QY, Zhao MG, Dai XY, Huang JH, Li LH, et al Aluminium-induced ethylene production is associated with inhibition of root elongation in *Lotus japonicus* L. Plant Cell Physiol. 2007; 48: 1229–1335. 17573361 10.1093/pcp/pcm077

[64] Sun P, Tian QY, Chen J, Zhang WH. Aluminium-induced inhibition of root elongation in *Arabidopsis* is mediated by ethylene and auxin. J Exp Bot. 2010; 61: 347–356. 10.1093/jxb/erp306 19858117 PMC2803203

[65] Yamamoto Y, Kobayashi Y, Matsumoto H. Lipid peroxidation is an early symptom triggered by Aluminium, but not the primary cause of elongation inhibition in pea roots. Plant Physiol. 2001; 125: 199–208. 11154329 10.1104/pp.125.1.199 PMC61002

[66] Basu U, Good AG, Taylor GJ. Transgenic *Brassica napus* plants overexpressing aluminium–induced mitochondrial manganese superoxide dismutase cDNA are resistant to aluminium. Plant Cell Environ. 2001; 24: 1269–1278.

[67] Miller G, Shulaev V, Mittler R. Reactive oxygen signaling and abiotic stress. Physiol Plant. 2008; 133: 481–489. 10.1111/j.1399-3054.2008.01090.x 18346071

[68] Sharma P, Dubey RS. Involvement of oxidative stress and role of antioxidative defense system in growing rice seedlings exposed to toxic concentrations of aluminium. Plant Cell Rep. 2007; 26: 2027–2038. 17653721 10.1007/s00299-007-0416-6

[69] Boscolo PRS, Menossi M, Jorge RA. Al–induced oxidadtive stress in maize. Phytochemistry. 2003; 62: 181–189. 12482454 10.1016/s0031-9422(02)00491-0

[70] Dipierro N, Mondelli D, Paciolla C, Brunetti G, Dipierro S. Changes in the ascorbate system in the response of pumpkin (*Cucurbita pepo* L.) roots to aluminium stress. J Plant Physiol. 2005; 162: 529–536. 15940870 10.1016/j.jplph.2004.06.008

[71] Singh S, Verma A, Dubey VK. Effectivity of anti–oxidative enzymatic system on diminishing the oxidative stress induced by aluminium in chickpea (*Cicer arietinum* L) seedlings. Braz J Plant Physiol. 2012; 24: 47–54.

[72] Malecka A, Jarmuszkiewicz V, Tomaszewska B. Antioxidative defense to lead stress in subcellular compartments of pea root cells. Acta Biochimica Polonica. 2001; 3: 687–698. 11833777

[73] Zhen Y, Miao L, Su J, Liua SH, Yina YL, Wanga SS, et al Differential responses of anti–oxidative enzymes to Aluminium stress in tolerant and sensitive soybean genotypes. J Plant Nut. 2009; 32: 1255–1270.

[74] Garzón T, Gunsé B, Moreno AR, Tomos AD, Barceló J, Poschenrieder C. Aluminium-induced alteration of ion homeostasis in root tip vacuoles of two maize varieties differing in Al tolerance. Plant Sci. 2011; 180: 709–715. 10.1016/j.plantsci.2011.01.022 21421422

[75] Inostroza-Blancheteau C, Rengel Z, Alberdi M, de la Luz Mora M, Aquea F, Arce-Johnson P, et al Molecular and physiological strategies to increase Aluminium resistance in plants. Mol Biol Rep. 2012; 39: 2069–2079. 10.1007/s11033-011-0954-4 21660471

[76] Wang P, Bi S, Ma L, Han W. Aluminium tolerance of two wheat cultivars (Brevor and Atlas66) in relation to their rhizosphere pH and organic acids exuded from roots. J Agric Food Chem. 2006; 54: 10033–10039. 17177538 10.1021/jf0611769

[77] Maron LG, Piñeros MA, Guimarães CT, Magalhaes JV, Pleiman JK, Mao C, et al Two functionally distinct members of the MATE (multi-drug and toxic compound extrusion) family of transporters potentially underlie two major Aluminium tolerance QTLs in maize. Plant J. 2010; 61: 728–740. 10.1111/j.1365-313X.2009.04103.x 20003133

[78] Ryan PR, Delhaize E, Jone DL. Function and mechanism of organic anion exudation from plant roots. Annu Rev Plant Physiol Plant Mol Biol. 2001; 52: 527–560. 11337408 10.1146/annurev.arplant.52.1.527

[79] Li XF, Ma JF, Matsumoto H. Pattern of Aluminium-induced secretion of organic acids differs between rye and wheat. Plant Physiol. 2000; 123(4): 1537–1544. 10938369 10.1104/pp.123.4.1537 PMC59110

[80] Ma BH, Wan JM, Shen ZG. H_2_O_2_ production and antioxidant responses in seeds and early seedling of two different rice varieties exposed to Aluminium. Plant Growth Regul. 2007; 52: 91–100.

[81] Ma JF, Zheng SJ, Matsumoto H, Hiradate S. Detoxifying aluminium with buckwheat. Nature. 1997; 390: 569–570.9403684

[82] Yang ZM, Sivaguru M, Horst WJ, Matsumoto H. Aluminium tolerance is achieved by exudation of citric acid from roots of soybean (*Glycine max*). Physiol Plantarum. 2000; 110: 72–77.

[83] Shen H, Yan X, Cai K, Matsumoto H. Differential Al resistance and citrate secretion in the tap and basal roots of common bean seedlings. Physiol Plantarum. 2004; 121(4): 595–603.

[84] Fan WH, Lou Q, Gong YL, Liu MY, Wang ZQ, Yang JL, et al Identification of early Al-responsive genes in rice bean (*Vigna umbellata*) roots provides new clues to molecular mechanisms of Al toxicity and tolerance. Plant Cell Environ. 2014; 37: 1586–1597. 10.1111/pce.12258 24372448

