# Supplementary figures and images for: Molecular Scanning and Morpho-Physiological Dissection of Component Mechanism in Lens Species in Response to Aluminium Stress
Source: PLoS One. 2016 Jul 28;11(7):e0160073. doi: 10.1371/journal.pone.0160073 (PMC4970855; doi:10.1371/journal.pone.0160073)

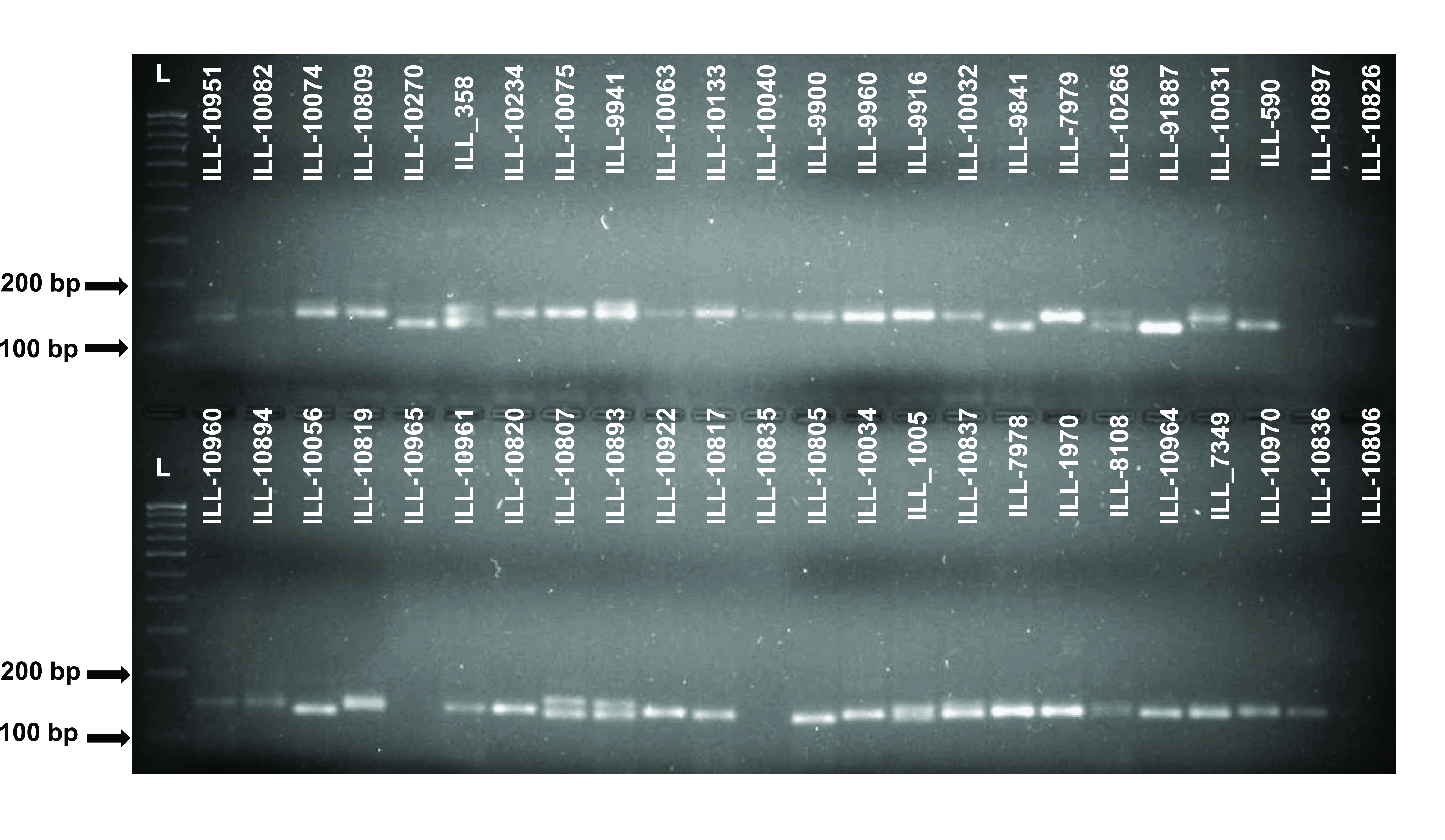

Supplement: S1 Fig — Base pairs (bp), 100bp DNA Ladder (L). (TIF) [file pone.0160073.s001.tif]

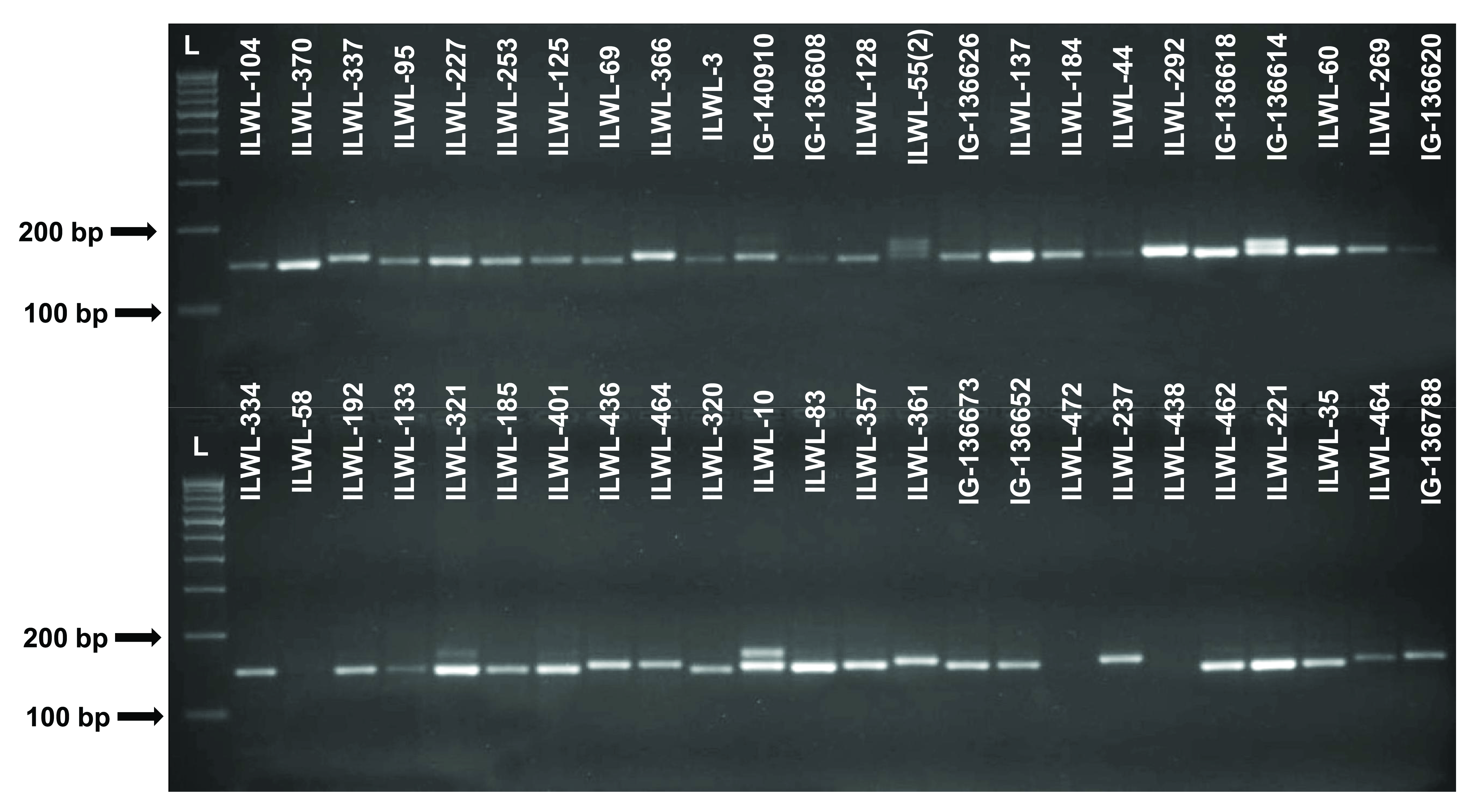

Supplement: S2 Fig — Base pairs (bp), 100bp DNA Ladder (L). (TIF) [file pone.0160073.s002.tif]

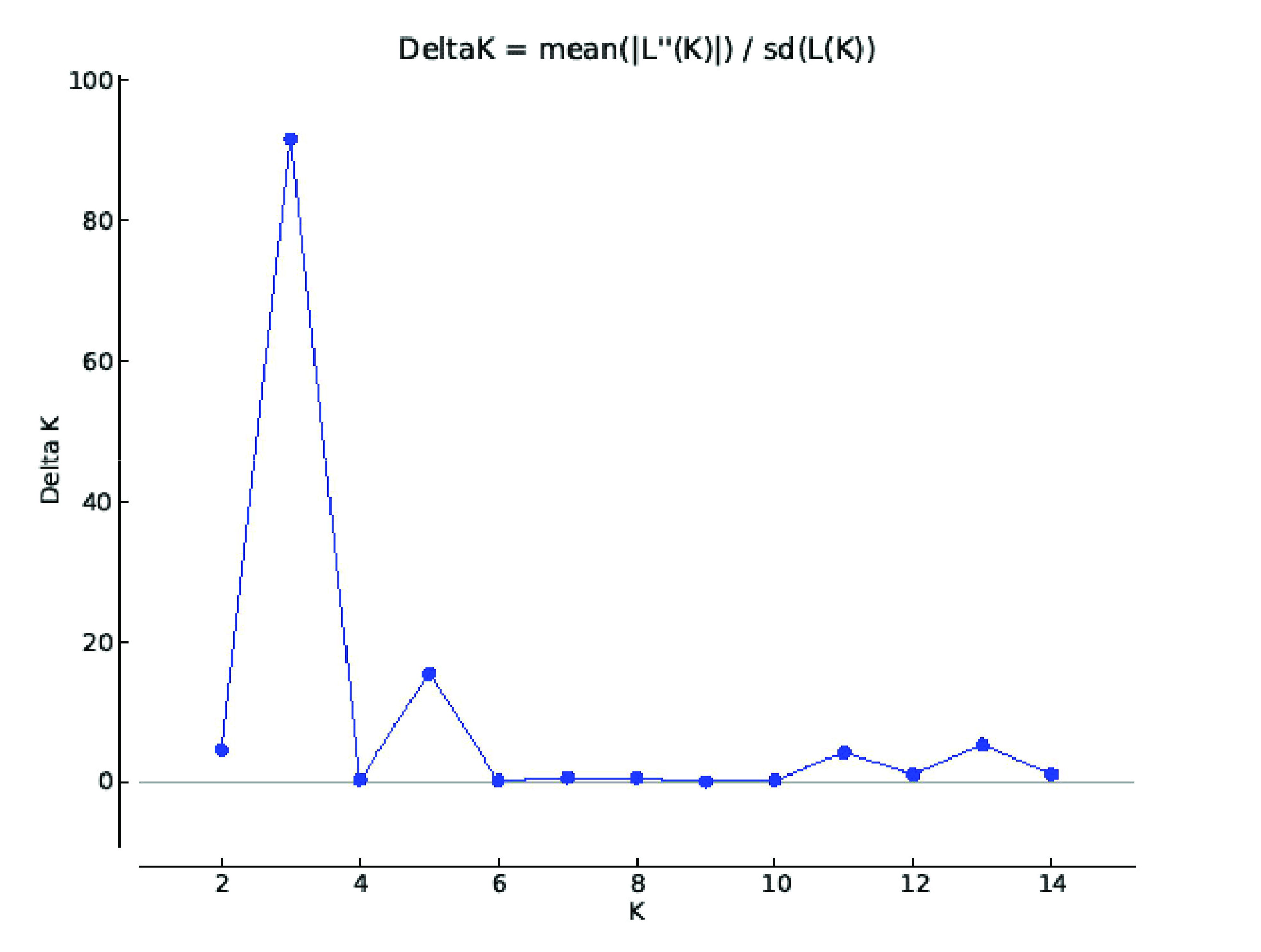

Supplement: S3 Fig — (TIF) [file pone.0160073.s003.tif]

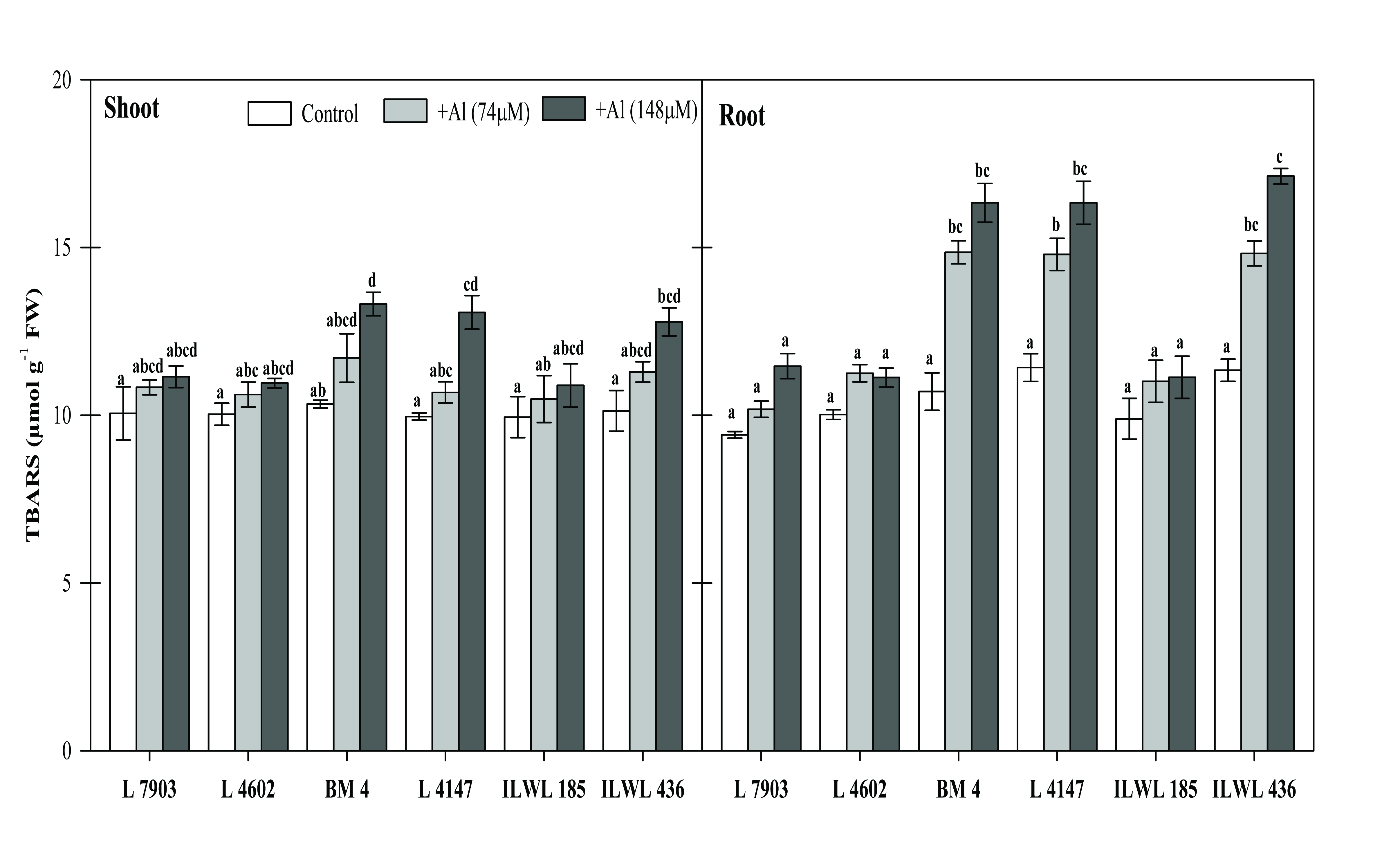

Supplement: S4 Fig — Means with the same small letters for each part of the plant do not statistically differ by the Tukey test at P≤0.05. (TIF) [file pone.0160073.s004.tif]

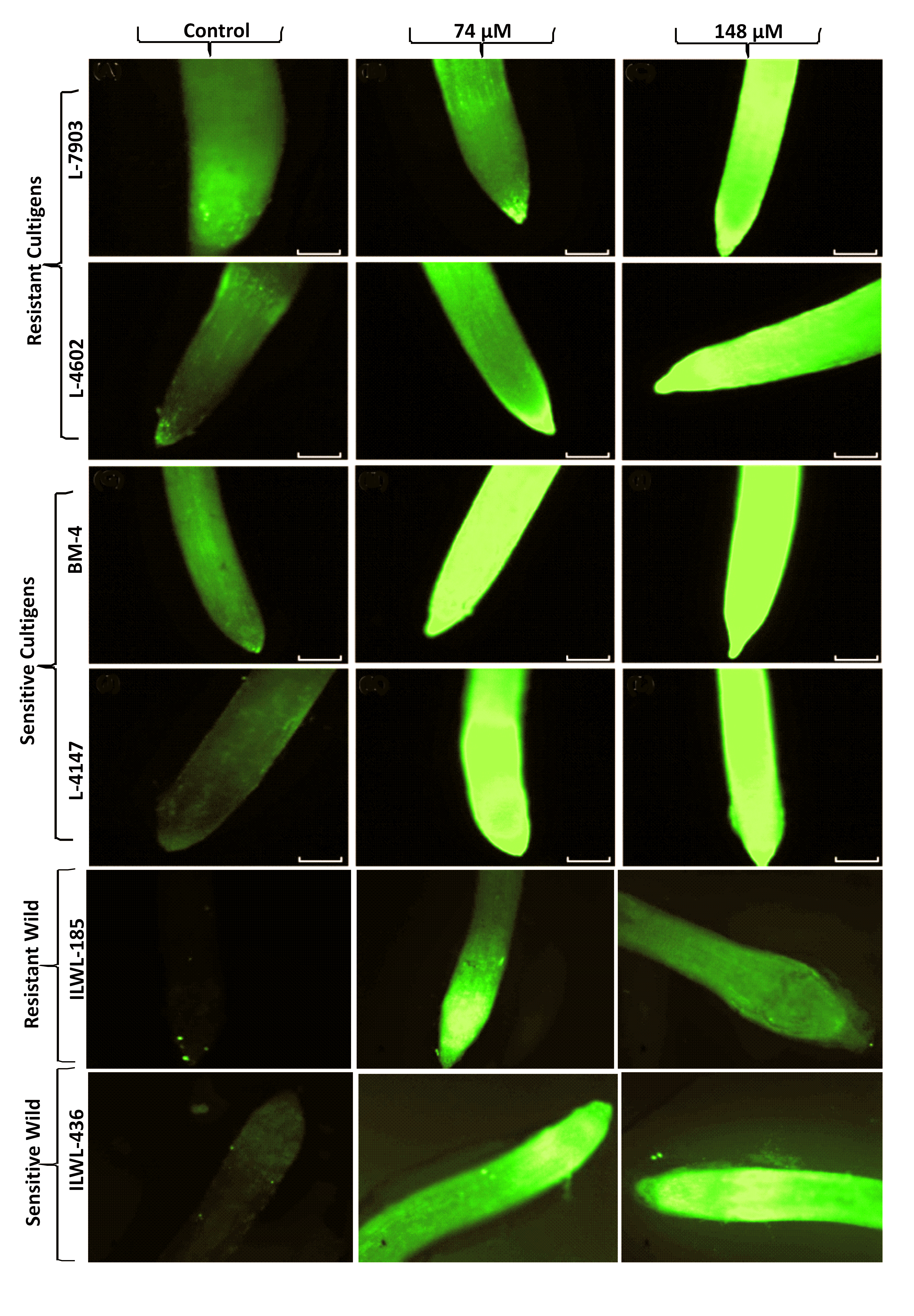

Supplement: S5 Fig — Bar in each figure represent 1 mm. (TIF) [file pone.0160073.s005.tif]
